# Genes for the Major Structural Components of Thermotogales Species’ Togas Revealed by Proteomic and Evolutionary Analyses of OmpA and OmpB Homologs

**DOI:** 10.1371/journal.pone.0040236

**Published:** 2012-06-29

**Authors:** Amanda K. Petrus, Kristen S. Swithers, Chaman Ranjit, Si Wu, Heather M. Brewer, J. Peter Gogarten, Ljiljana Pasa-Tolic, Kenneth M. Noll

**Affiliations:** 1 Department of Molecular and Cell Biology, University of Connecticut, Storrs, Connecticut, United States of America; 2 Environmental Molecular Sciences Laboratory, Pacific Northwest National Laboratory, Richmond, Washington, United States of America; J. Craig Venter Institute, United States of America

## Abstract

The unifying structural characteristic of members of the bacterial order Thermotogales is their toga, an unusual cell envelope that includes a loose-fitting sheath around each cell. Only two toga-associated structural proteins have been purified and characterized in *Thermotoga maritima*: the anchor protein OmpA1 (or Ompα) and the porin OmpB (or Ompβ). The gene encoding OmpA1 (*ompA1*) was cloned and sequenced and later assigned to TM0477 in the genome sequence, but because no peptide sequence was available for OmpB, its gene (*ompB*) was not annotated. We identified six porin candidates in the genome sequence of *T. maritima*. Of these candidates, only one, encoded by TM0476, has all the characteristics reported for OmpB and characteristics expected of a porin including predominant β-sheet structure, a carboxy terminus porin anchoring motif, and a porin-specific amino acid composition. We highly enriched a toga fraction of cells for OmpB by sucrose gradient centrifugation and hydroxyapatite chromatography and analyzed it by LC/MS/MS. We found that the only porin candidate that it contained was the TM0476 product. This cell fraction also had β-sheet character as determined by circular dichroism, consistent with its enrichment for OmpB. We conclude that TM0476 encodes OmpB. A phylogenetic analysis of OmpB found orthologs encoded in syntenic locations in the genomes of all but two Thermotogales species. Those without orthologs have putative isofunctional genes in their place. Phylogenetic analyses of OmpA1 revealed that each species of the Thermotogales has one or two OmpA homologs. *T. maritima* has two OmpA homologs, encoded by *ompA1* (TM0477) and *ompA2* (TM1729), both of which were found in the toga protein-enriched cell extracts. These annotations of the genes encoding toga structural proteins will guide future examinations of the structure and function of this unusual lineage-defining cell sheath.

## Introduction

The Thermotogales is an order of bacteria defined by a unique outer envelope called the “toga” [1]. This unconventional structure balloons out at the poles of the cells forming a pronounced periplasmic space [2], [3]. This envelope not only serves as a barrier to the external milieu, but also provides a structure for the organization of polysaccharide hydrolases exposed on the cell surface, allowing the utilization of insoluble carbon sources [4], [5].

Although *Thermotoga maritima* was the first hyperthermophilic bacterium discovered, very little is known about the composition of its toga [5]. Of the hydrolytic enzymes isolated from *T. maritima*, an amylase (AmyA, TM1840) and a xylanase (XynA, TM0061) have been definitively associated with the toga [4], [5]. Only two other proteins, OmpA1 (previously called Ompα) [6], [7] and OmpB (previously called Ompβ) [3], have been conclusively found to be within the toga fraction.

The chemical nature and protein composition of the outer envelope, or toga, of species of the Thermotogales remains poorly studied. Since these organisms stain Gram-negative, it is commonly concluded that they must have features of Gram-negative proteobacteria like *Escherichia coli*. However, the Gram reaction only provides general information about the character of cell envelopes, and does not provide sufficient clues about the chemical or protein composition of organisms distantly related to the proteobacteria to allow direct comparisons to be made between members of such lineages [8], [9]. The problem is exacerbated when the nomenclature of envelope elements used with different organisms overlaps, but does not correspond, as with *E. coli* and *T. maritima*.

In *E. coli*, the OmpA porin protein is one element that links the outer membrane to the peptidoglycan [10]. It is embedded in the outer membrane by N-terminal antiparallel β-strands and has a C-terminal periplasmic domain bound to the peptidoglycan, apparently in a non-covalent manner [11].

In *T. maritima*, OmpA1 plays a physiological role similar to that of the *E. coli* OmpA except that OmpA1 is not a porin. Though they have similar names, the two proteins are neither evolutionarily nor structurally related. *T. maritima* OmpA1 is a 45.3 kDa rod-shaped spacer protein that serves to anchor the toga to the cell body, probably at the peptidoglycan layer [6], [7]. Its N-terminus contains an S-layer homology (SLH) domain that likely anchors it to the peptidoglycan or a similar saccharide layer in the envelope [6], [7]. A 316 amino acid alpha helical coiled coil region follows that serves to separate the toga from the cytoplasmic membrane by ∼50 nm, except where the toga balloons away from the membrane. OmpA1 appears to remain associated with OmpB in the portion of the toga that peels away from the cytoplasmic aspect of cells during growth. A C-terminus of ∼20 hydrophobic amino acids may anchor OmpA1 in the toga.

*T. maritima* OmpB was described as a 42 kDa porin that likely constitutes the largest fraction of the toga protein content [3]. Freeze etching images of the surface of *T. maritima* cells showed triangular orifices regularly arrayed across the entire surface of the toga with a p3 lattice of 11 nm, a motif and dimension common to *E. coli* porins [3]. Imaging of purified cell sheaths showed that OmpB forms trimers very similar to those of the *E. coli* porin OmpF. *E. coli* has no OmpB protein. Instead *ompB* designates a genetic locus that encodes OmpR and EnvZ, two proteins that control expression of the porins OmpC and OmpF in response to different environmental signals.

To date, only three toga proteins (the amylase TM1840, xylanase TM0061 and OmpA1 TM0477) have been successfully annotated in the *T. maritima* genome sequence through cloning and sequencing of their genes [4]–[6], [12], [13]. Using N-terminal sequence derived from the purified native OmpA1 protein, its gene was cloned and sequenced [6], [7] so that when the *T. maritima* genome was sequenced the ORF encoding OmpA1 could be assigned [14]. OmpA1 is encoded by TM0477 in the *T. maritima* genome sequence. OmpB, the major constituent of the toga, was purified in 1990, but no amino acid or nucleotide sequence data were able to be gathered [3], so the gene encoding it has yet to be identified in the genome sequence. This lack of annotation has hampered studies of the evolution of toga proteins and comparative analyses of toga proteins across the Thermotogales lineage.

We sought to identify the gene encoding OmpB by sequencing the proteins present in purified toga-sheath fractions. We sought further support for the gene assignment using bioinformatic structural and phylogenetic analyses of the OmpB candidates in the genome sequences of *T. maritima* and its relatives. This examination produced a catalog of toga-associated proteins, identified the gene encoding OmpB, and revealed a new class of OmpA homologs.

## Results

### The *T. maritima* Genome Sequence has Six Porin Candidates

The genome sequence of *T. maritima* was examined for genes likely to encode the OmpB porin [14]. Six porin candidates were identified by meeting at least three of the following criteria: (1) the reported size of OmpB (approximately 42 kDa), (2) a signal sequence to allow export, (3) likely β-sheet content within the range of known porins, (4) globularity, and (5) a characteristic porin carboxy terminus (see description below) (Table 1). These loci were TM0153, TM0354, TM0476, TM0639, TM0642, and TM1274.

**Table 1:** Characteristics of porin candidates from *T. maritima* as compared to those of known porins.

Protein/ORF*	Molecular Weight (kD)	Signal peptide	β Strand content (%)‡	Globularity	Porin C-terminus
OmpC*^E^*	40.38	+	29.16/33	+	+
OmpF*^Ec^*	39.36	+	28.45/33	+	+
PhoE*^Ec^*	38.93	+	23.93/36	+	+
ScrY*^Se^*	55.11	+	18.42/22	+	+
ScrY*^Ye^*	56.70	+	17.77/24	+	+
TM0153	70.74	–	23.11/23	+	+
TM0354	49.11	+	12.14/15	+	–
TM0476	44.90	+	26.07/22	+	+
TM0639	45.60	+	35.00/30	+	–
TM0642	29.63	–	29.06/23	+	+
TM1274	27.09	+	43.27/38	+	+

Characteristics are defined in the text. **Legend:** +, protein is predicted to possesses the attribute; –, protein is predicted to not possess the attribute.

* Proteins are *Escherichia coli* OmpC, OmpF, PhoE; *Salmonella enterica* ScrY; and *Yersinia enterocolitica* ScrY. ORFs are from TM, *T. maritima*. ‡ Percent β strand content calculated utilizing the multisequence alignment programs STRAP and SCRATCH, respectively.

To compare the structural characteristics of the candidate proteins with those of known porins, the *T. maritima* porin candidates and five bacterial porin sequences were analyzed using STRAP and SCRATCH protein prediction software. The bacterial porins were found to have β-strand contents ranging from 17.77–29.16% (STRAP) and 22–36% (SCRATCH) (Table 1). TM0476 has a calculated β-strand content of 26.07% and 22%, falling within both ranges. Though the β-strand contents of candidates TM0153 and TM0642 also fall within both ranges, they each lack a signal peptide and are not the appropriate molecular weight for OmpB (Table 1).

Only the product of TM0476 possesses all the expected characteristics of a porin. The product of TM0476 is a 417 amino acid protein, with a molecular weight of 44.87 kDa. Its sequence contains a signal peptide marking the protein for export. Other characteristics of the TM0476 product are consistent with it being OmpB. It is predicted to be globular, suggesting the possibility for oligomerization.

The location of TM0476 in the genome is also consistent with this assignment. TM0476 is adjacent to *ompA1* (TM0477), the ORF encoding OmpA1, and is transcribed in the same direction. These genes appear to be in an operon, as shown by the ProOpDB database [15]. Together these characteristics support the identification of TM0476 as the gene encoding OmpB (*ompB*).

### The Amino Acid Sequence and Composition of OmpB (TM0476) Showed Features of Known Porins

As porins possess little sequence similarity across species, they are difficult to identify using sequence alignment comparisons [16], but conserved characteristic sequence features can be utilized to predict porin identities. Amino acid composition, trimeric channel structure, N- and C-terminal sequences, and amino acid content are conserved across large evolutionary distances. A C-terminal phenylalanine residue is essential for the anchoring of porins to the cell surface [17], [18]. However, *E. coli* OmpA is an exception as it lacks this feature and its C-terminus is in the periplasm [17], [18]. The last ten C-terminal amino acids of typical porins contain hydrophobic residues at positions 1, 3, 5, 7 and 9 and these are also essential for anchoring them in the outer membrane [17], [18]. A comparison of the C-terminal amino acids of OmpB with known porins shows that it has a terminal phenylalanine as well as hydrophobic residues at positions 1, 3, 5, 7, and 9 (Table 2). A hydropathy plot of the sequence of OmpB (not shown) shows its C-terminus forms a transmembrane hydrophobic anchor like that of Gram-negative bacterial porins which have amphipathic beta strands composed of hydrophobic residues at C-terminal positions 1, 3, 5, 7, and 9 [17], [18].

**Table 2:** The C-terminal amino acids of TM0476 are like those of several confirmed porins.

	C-terminal amino acids (N → C)									
Protein	10	9	8	7	6	5	4	3	2	1
TM0476 (*T. maritima*)	Y	**L**	Y	**L**	K	**A**	S	**V**	A	**F**
PhoE (*S. enterica*)	I	**V**	A	**I**	G	**L**	T	**Y**	Q	**F**
PhoE (*E. coli*)	I	**V**	A	**V**	G	**M**	T	**Y**	Q	**F**
OmpF (*E. coli*)	T	**V**	A	**V**	G	**I**	V	**Y**	Q	**F**
OmpC (*E. coli*)	I	**V**	A	**L**	G	**L**	V	**Y**	Q	**F**

Position 1 indicates the C-terminal amino acid. Hydrophobic residues are boldface. All porins shown possess the essential terminal phenylalanine and hydrophobic residues at positions 3, 5, 7 and 9.

Porins also have a distinct amino acid composition with low percentages of Arg, Cys, Glu, His, Ile, Met, Pro, and Trp and high percentages of Ala, Gly, Asn, Asp, and Leu [16]. TM0476 has low levels of Cys, Pro, His and Met and high levels of Asn, Asp, and Gly. The composition of OmpB of *T. maritima* is comparable to that of OmpC and OmpF of *E. coli*.

### Putative OmpB was Identified within Toga Fractions Collected by Sucrose Gradient Ultracentrifugation

To experimentally demonstrate which ORF is *ompB*, we set out to purify the native porin protein described by Rachel et al. [3] using comparable methods and then determine the protein’s sequence to identify its gene in the genome sequence. Rachel et al. reported that toga sheath material can be collected by gradient centrifugation and that the resulting protein fractions contain protein complexes with apparent M_r_ values >100 kDa as determined by SDS gel electrophoresis [3]. These oligomers were identified as OmpA1 and OmpB and were found to dissociate into their respective monomers of 40–45 kDa after heat treatment (for an unreported amount of time) in 2% SDS prior to SDS-PAGE [3].

To obtain toga proteins, we resolved a membrane pellet using sucrose density gradient ultracentrifugation. SDS-PAGE analysis of fractions collected from the sucrose gradient displayed several protein bands in the 50–65% sucrose fractions (the “toga fractions,” fractions 15–21) including bands within the range of interest (100 to 250 kDa) (not shown).

A representative sucrose gradient fraction from the “toga fractions,” fraction 18, was selected to locate OmpB within the outer envelope fraction. Fraction 18 was resolved by SDS-PAGE and nine bands were excised and submitted for LC/MS/MS analysis (Fig. S1). A large number of proteins of different sizes were detected in each band, a result not surprising for LC/MS/MS analyses of gel bands as discussed in more detail below. In addition, contrary to the observations of Rachel, et al [3], we observed that our envelope protein preparations were incompletely denatured by boiling the samples in 2.5% SDS for 5 min, so that a number of proteins apparently co-migrated on SDS gels. The putative OmpB (TM0476) was detected in this sample. Experimentally demonstrated toga proteins including OmpA1 (TM0477) and XynA (TM0061) were also present in the bands providing proof that the predicted OmpB, TM0476, is located in the outer envelope.

### An OmpB-like Trimer was Further Purified from Enriched Toga Material by Hydroxyapatite Chromatography

The presumed toga fractions (the 50–65% sucrose fractions 15–21) were pooled for further purification of OmpB by hydroxyapatite chromatography. OmpB has been shown to possess an affinity for hydroxyapatite, with the OmpB porin eluting at approximately 200 mM phosphate [2], [6]. Fig. S2, panel A shows a preparative SDS-PAGE analysis of fractions eluted from a hydroxyapatite column loaded with the pooled sucrose density gradient toga fractions 15–21. Fractions 6–8 of the hydroxyapatite column eluate contain a band of approximately150 kDa that is relatively isolated and this size is consistent with the elution characteristics of the trimer form of OmpB [3]. When the column fractions were treated with stronger denaturing conditions [1% octyl-polyoxyethyleneoctyl-POE (octyl-POE) and 2.5% SDS at 100°C for 10 min] prior to loading on the SDS-PAGE gel, many of the high molecular weight bands disappeared and in lanes 6′ and 7′ a single, faint low molecular weight band was apparent (Fig. S2, panel B). A similar band was reported in the initial characterization of OmpB [3], [6]. Proteomic analysis of these bands revealed peptide matches to TM0477 (OmpA1) in the band from lane 6′ and TM0476 (OmpB) in the band from lane 7′ (Table S2).

The SDS gel bands clearly contain other proteins, some of which are not likely in the toga, but these results are common for proteomic analyses of gel bands. Proteins adhering to one another likely account for the presence of cytoplasmic and cell membrane proteins in these preparations. Membrane-associated proteins from thermophiles are more generally resistant to denaturation by detergents and heat, so *T. maritima* membrane proteins like OmpB might be particularly prone to associating with other proteins even under what appear to be harsh denaturing conditions. As a rule, the intensity of a mass spectrometric signal cannot easily be correlated with the amount of a protein present a sample [19], so the number of peptides observed for each protein is not necessarily a measure of its relative abundance. TM0476 is the only protein in these fractions that meets the description of OmpB and is a porin candidate (Table 1), so we conclude that TM0476 is *ompB*, the gene encoding the major toga porin of *T. maritima*.

### The Purified OmpB had β-sheet Content as Determined by Circular Dichroism

A hydroxyapatite fraction enriched in OmpB was analyzed by circular dichroism (CD) for β-sheet secondary structure information. The CD spectrum of this fraction showed predominantly β-like characteristics, possessing a negative band at approximately 215 and lacking a distinct α-helix negative band at 222 nm (Fig. S3) [20]. The spectrum also lacked the distinct positive peak at 212 nm that would indicate a predominantly random coil. Thus this fraction is composed of protein with predominantly β-sheet composition, an observation consistent with previous observations of *T. maritima* OmpB that, like other porins, has high β-sheet content [3].

### Phylogenetic Analyses Revealed Likely *ompA* and *ompB* Homologs among Thermotogales Species

Since OmpA1 and OmpB are the two dominant structural proteins of the *T. maritima* toga and all known species of the Thermotogales have togas, we examined the genome sequences of other members of the Thermotogales to identify possible homologs of these proteins. OmpB homologs were found using a PSI-BLAST search (see Methods) and are present in all species except *Thermosipho africanus* and *Thermotoga lettingae*. A distant homolog to OmpB was detected in *Vibrio mimicus* with an E value of 8E-6 (using the *T. maritima* OmpB sequence in a BLASTp query). Currently there are no more similar sequences outside the Thermotogales in the GenBank nr database suggesting that OmpB-like proteins are unique to the Thermotogales.

**Figure 1:**
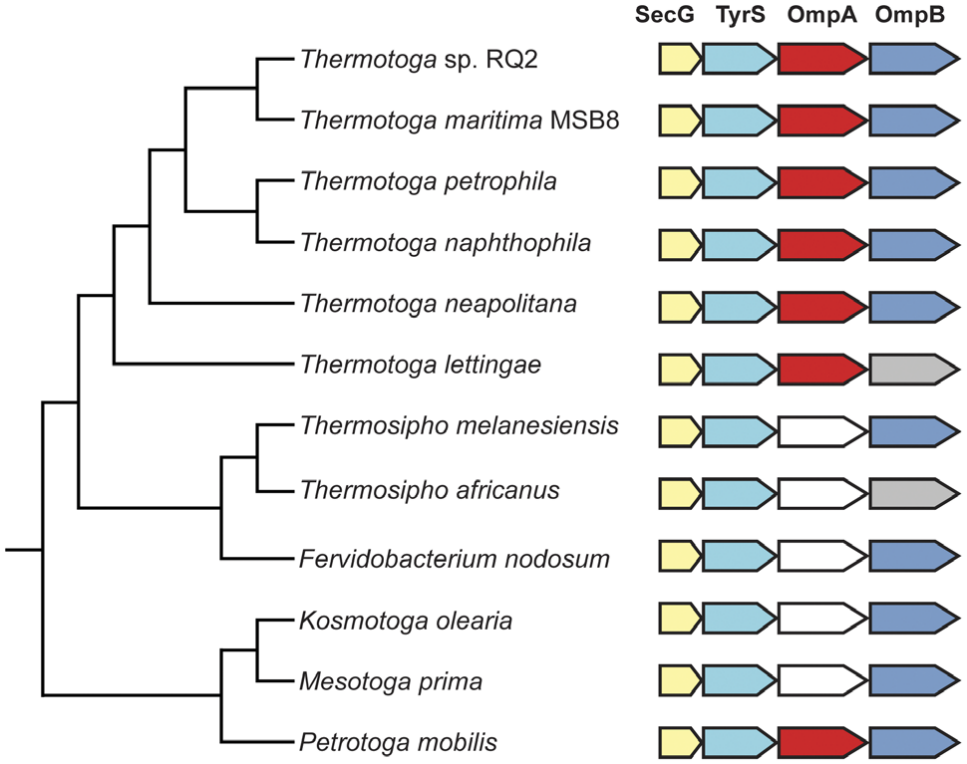
Syntenic regions containing *T. maritima* OmpA1 and OmpB homologs and putative analogs mapped onto an rRNA gene reference tree. The coloring of the individual genes indicates whether the gene is a homolog, paralog, or putative analog. *segG* homologs are yellow, *tyrS* homologs are light blue, *ompA1* homologues are red, and *ompB* homologues are dark blue. *ompA* paralogs are white (see Fig. 2). *ompB* putative analogs are grey. The tree is a concatenated 23S-16S rRNA gene cladogram. Branch lengths do not reflect the extent of divergence.

The genomic regions encoding OmpB and OmpA are very similar in Thermotogales species. These genes are present in a syntenic region with two other genes: *secG*-*tyrS*-*ompA*-*ompB* (Fig. 1). Several species have paralogs of *ompA1* (examined in detail below) and two species, *T. lettingae* and *Thermosipho africanus*, have genes downstream of *ompA* that do not show significant to similarity to TM0476 in a PSI-blast search (see Methods). These two non-orthologous genes downstream of the ompA genes are currently annotated as encoding hypothetical proteins in these two genome sequences. Given the conserved gene order, it is likely that these genes are either analogous porins, or homologs that have diverged beyond recognition. To test this hypothesis, we examined the sequences of the OmpB orthologs and these putative analogs for porin characteristics (Table 3). Though they lack the globularity seen in most of the orthologous proteins, the putative analogs have many of the same porin-like characteristics shared by the orthologs. In addition, they also have C-terminal amino acid compositions like those of TM0476 and known porins, including a terminal phenylalanine (Table 4). Consequently, although these proteins are not recognizable homologs of *T. maritima* OmpB and its orthologs, these proteins likely serve a similar function.

**Table 3:** Characteristics of *T. maritima* OmpB orthologs and putative syntenic analogs in Thermotogales species.

Protein/ORF*	Molecular Weight (kD)	Signal peptide	β Strand content (%)‡	Globularity	Porin C-terminus
TM0476	44.90	+	26.07/22	+	+
TRQ2_0459	45.53	+	34.62/26.39	+	+
Tpet_0444	45.04	+	26.14/23.5	+	+
Tnap_0258	45.04	+	26.14/23.5	+	+
CTN_0196	44.18	+	25.3/23.6	+	+
Tmel_0175	42.76	+	24.4/38.65	+	+
Fnod_1725	47.99	+	36.97/35.19	+	+
Kole_1501	38.70	+	30.08/29	-	+
Theba_0319	42.71	+	28.12/26	-	+
Pmob_0056	47.88	+	27.48/27	+	+
Tlet_1718	35.86	+	31.25/21	-	+
THA_406	32.43	+	42.76/43	-	+

Characteristics are defined in the text. **Legend:** +, protein is predicted to possesses the attribute; –, protein is predicted to not possess the attribute.

ORFs are from TM, T. maritima; TRQ2, Thermotoga species strain RQ2; Tpet, Thermotoga petrophila; Tnap, Thermotoga naphthophila; CTN, Thermotoga neapolitana; Tmel, Thermotoga melanesiensis; Fnod, Fervidobacterium nodosum; Tlet, Thermotoga lettingae; THA, Thermosipho africanus; Kole, Kosmotoga olearia; Pmob, Petrotoga mobilis; and Theba, Thermotogales bacterium mesG1.Ag.4.2 (Mesotoga prima). ORFs above the line are homologs of TM0476, those below the line are possible analogs of TM0476.

‡ Values of the percent β strand content were calculated utilizing the multisequence alignment programs STRAP and SCRATCH, respectively.

**Table 4:** The C-terminal amino acids of the OmpB orthologs and putative analogs show porin characters.

	C-terminal amino acids (N → C)									
Protein	10	9	8	7	6	5	4	3	2	1
TM0476	Y	**L**	Y	**L**	K	**A**	S	**V**	**A**	**F**
TRQ2_0459	**F**	G	**F**	**I**	T	Y	R	**L**	**A**	**F**
Tpet_0444	Y	**L**	Y	**L**	K	**A**	S	**V**	**A**	**F**
Tnap_0258	Y	**L**	Y	**L**	K	**A**	S	**V**	**A**	**F**
CTN_0196	Y	**L**	Y	**L**	K	**A**	E	**V**	E	**F**
Tmel_0175	Y	**A**	K	**L**	S	**W**	S	**V**	S	**F**
Fnod_1725	N	**L**	K	**L**	T	Y	S	**A**	S	**F**
Kole_1501	Y	**A**	Y	**V**	G	Y	Y	**A**	**A**	**F**
Theba_0319	S	**L**	G	**L**	Y	**F**	D	K	Y	**F**
Pmob_0056	Y	**L**	Y	**L**	K	**A**	E	**F**	K	**F**
Tlet_1718	T	**L**	**A**	**W**	R	**M**	R	**V**	Y	**F**
THA_406	T	**L**	N	**M**	N	**A**	H	**F**	D	**F**

Position 1 indicates the C-terminal amino acid. Hydrophobic residues are boldface. All porins shown possess the essential terminal phenylalanine and most have hydrophobic residues at positions 3, 5, 7 and 9. ORFs above the line are homologs of TM0476, those below the line are possible analogs of TM0476.

**Figure 2:**
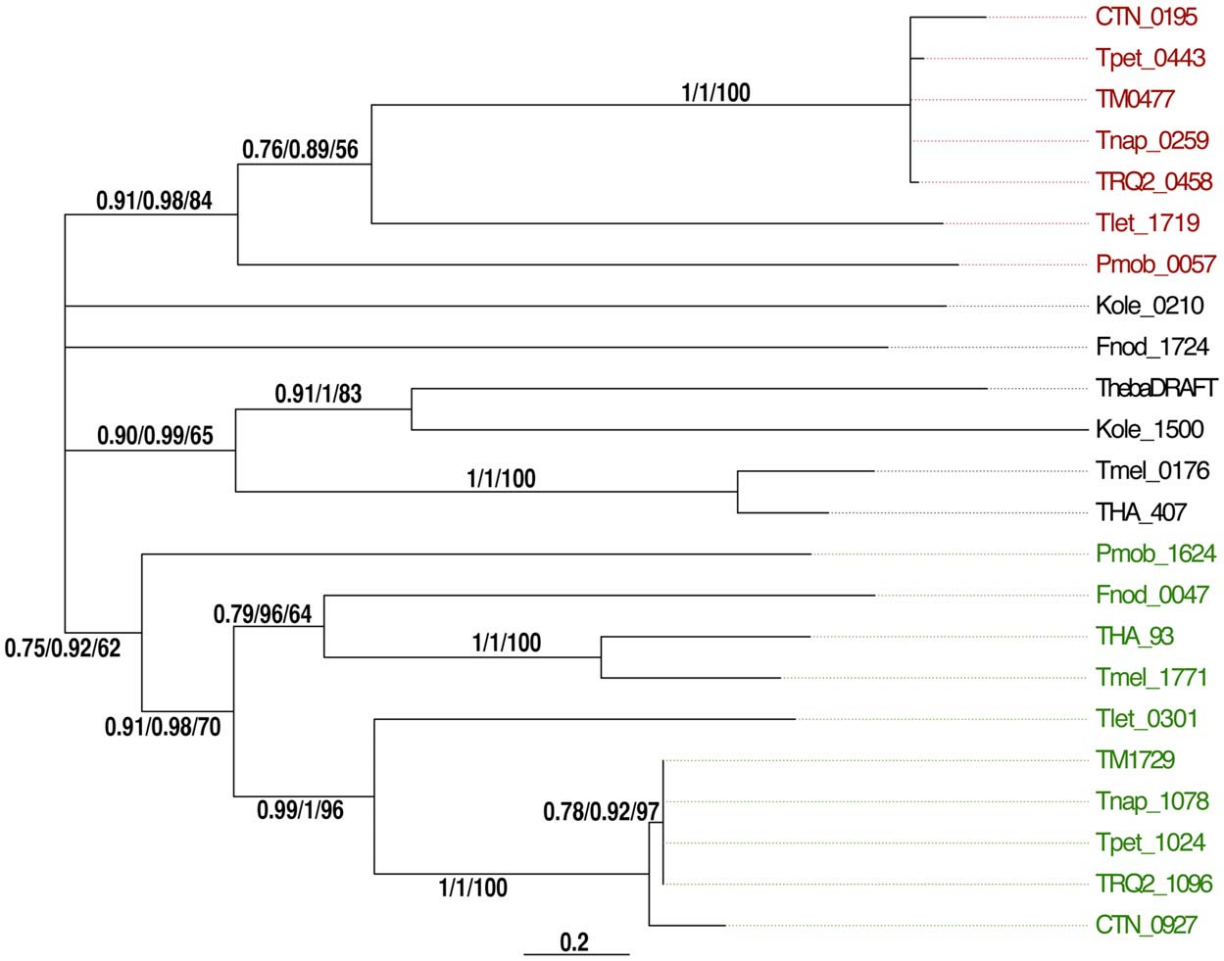
Maximum likelihood phylogenetic tree of OmpA protein sequences from several Thermotogales species. Red labels indicate likely *T. maritima* OmpA1 (TM0477) orthologs, green labels indicate likely OmpA2 (TM1729) orthologs, and homologs whose type of homology cannot be ascertained are labeled in black. The tree was calculated as unrooted phylogeny, but is depicted as rooted between the likely OmpA1 and OmpA2 clusters. Branches with approximate Likelihood Ratio Test (aLRT) support values ≤0.75 were collapsed. aLRT, posterior probability, and bootstrap support values are given above or below the branch to which they pertain. Organism abbreviations and gene identification numbers for loci are *Petrotoga mobilis* (Pmob_0057, 160901548 and Pmob_1624, 160903060); *T. lettingae* (Tlet_1719, 157364570 and Tlet_0301, 157363168); *T. petrophila* (Tpet_1024, 148270158 and Tpet_0443, 148269583); *T. maritima* (TM0477, 15643243 and TM1729, 15644475); *T. napthophila* (Tnap_1078, 281412500 and Tnap_0259, 281411698) *T. neapolitana* (CTN_0195, 222099169 and CTN_0927, 222099901); *Thermotoga*. sp. strain RQ2 (TRQ2_0458, 170288259 and TRQ2_1096, 170288887); *Fervidobacterium nodosum* (Fnod_1724, 154250391 and Fnod_0047, 154248750); *Kosmotoga olearia* (Kole_0210, 239616617 and Kole_1500, 239617873); *Thermosipho africanus* (THA_407, 217076525 and THA_93, 217076226); *Thermosipho melanesiensis* (Tmel_0176, 150020084 and Tmel_1771, 150021641); Thermotogales bacterium mesG.Ag.4 (*Mesotoga prima*) (ThebaDRAFT_0522, 307297745; now Theba_0318).

OmpA homologs are present in all Thermotogales species, but their relationships to one another are complex (Fig. 2). All the members of the Thermotogales have two *ompA* genes, except Thermotogales bacterium mesG.Ag.4 (now *Mesotoga prima* [21]), which has only one. Phylogenetic reconstructions reveal that there are two well supported paralogous clusters (indicated in red and green in Fig. 2), OmpA1 and OmpA2, and a third group of poorly supported OmpA homologs. The *T. maritima ompA2* is locus TM1729. Its product is the same size as OmpA1 and is predicted to be predominantly α-helical. This protein was identified in the toga fraction of cell extracts providing evidence that its gene is expressed (Table S1). Analyses of the sequences of all the OmpA homologs as described in Methods suggest that all have an SLH domain motif, are predicted to be over 75% α-helical, and have hydrophobic C-termini [6], [22]–[24]. These features suggest that all identified OmpA homologs could link the outer sheath to the peptidoglycan layer in their respective organisms.

## Discussion

We have identified the gene that encodes OmpB, the major porin of *Thermotoga maritima*. The *ompB* gene is locus TM0476 in the *T. maritima* genome sequence. TM0476 was annotated as a hypothetical protein, but of the other porin candidates in the genome sequence, its protein sequence has the most features consistent with the published characteristics of OmpB and of other porins. We found the protein encoded by TM0476 in the toga fraction of *T. maritima* cells and identified it by LC/MS/MS. We showed experimentally that this purified protein has β-sheet character as expected in a porin. Further evidence for our assignment is that the *ompB* gene is adjacent to the gene for the other major structural toga protein, OmpA1.

Since the toga is the defining characteristic of the Thermotogales, we expected to find homologs of *T. maritima* OmpB in all the sequenced genomes of the lineage. Surprisingly, two species did not have detectable homologs. *T. lettingae* and *Thermosipho africanus* instead have possible analogs of OmpB encoded downstream of their *ompA* genes. Those putative analogs have the characteristics of porins and likely serve a similar function. The OmpB proteins have only distantly related homologs outside the Thermotogales. Studies of the evolution of porins have largely been restricted to examinations within the Enteriobacteriaceae so it is not clear if the OmpBs’ evolutionary novelty is an unusual feature of Thermotogales porins or if all porin evolutionary families are only distantly related to one another. Porins in *Yersinia* species show evidence of positive selection [25], likely in response to host immune responses as well as phage infection and environmental factors. Perhaps the Thermotogales porins also respond to selective environmental factors and so have changed in sequence sufficient to erase traces of their ancestry. The problem of their evolutionary history is likely further complicated by the lack of genome sequences from closer relatives of the Thermotogales.

Given the essential structural role of the *T. maritima* anchor protein OmpA1, one might expect other Thermotogales species to have homologs proteins. All species do have such homologs, but surprisingly there are more OmpA-like genes in the lineage. *T. maritima* has a second OmpA, TM1729. TM1729, OmpA2, was detected in our toga fraction, therefore it is expressed. The sequence of OmpA2 suggests that it has the same structural features as OmpA1. All other Thermotogales species also have two OmpAs, except *M. prima* that has only one OmpA-like protein. Why *T. maritima* and the other species have a second OmpA-type protein is unknown.

Our results provide the first comprehensive examination of the protein composition of the toga and our analyses of the Omp proteins in the Thermotogales have revealed unexpected complexity and evolutionary histories. More detailed examinations are underway to learn how the toga forms and what roles these proteins play in its functions.

## Materials and Methods

### Cell Growth and Membrane Collection

*Thermotoga maritima* MSB8 (DSM 3109) was obtained from the Deutsche Sammlung von Mikroorganismen und Zellkulturen, Braunschweig, Germany. Cells were grown on a modified basal defined medium under anaerobic conditions with maltose as carbon source [26]. Cells were grown to stationary phase, collected by centrifugation at 6,000×g for 15 min, and washed three times with 100 mM ammonium bicarbonate buffer pH 8.0. Cells were lysed by bead beating (Zymo Research ZR Bashingbeads, 0.5 mm), followed by sonication on ice. Lysates were subjected to centrifugation at 100,000×g for 1 h and the resulting pellet washed twice with 100 mM ammonium bicarbonate. Cells harvested from one liter of dense cell culture yielded approximately 0.5 g wet weight of membrane fraction. The resulting membrane pellets were washed three times with 100 mM ammonium bicarbonate and stored at –20°C.

### Enrichment of Toga Sheaths

A 1.5 g membrane pellet was resuspended in 1 mL 100 mM ammonium bicarbonate and applied to a sucrose step gradient consisting of 30% (1.8 ml), 40% (2.4 ml), 50% (1.2 ml), 55% (1.2 ml) and 65% (0.6 ml) sucrose. The resulting gradient was centrifuged at 100,000×g for 18 h at 4°C. Twenty-four fractions were collected from top to bottom and each analyzed by SDS-PAGE. Fractions 15–21 had high molecular weight material characteristic of the toga fraction. Nine bands with molecular weights >60 kDa from an SDS-PAGE lane of a representative fraction, fraction 18, were excised (Fig. S1) and analyzed by LC/MS/MS.

### Purification of OmpB-like Trimer

Fractions 15–21 were pooled and concentrated using a 10 kDa cutoff centrifugal filtration device (Millipore). The concentrated sample was solubilized with 1% octyl-POE then loaded onto a 10 ml hydroxyapatite column, which was previously washed and equilibrated with 1 mM sodium phosphate, pH 7.3. The column was washed with 3 column volumes of 1 mM sodium phosphate until no further protein material eluted. Subsequent step-wise elution of bound protein was carried out with ten aliquots, of 0.5 ml each, ranging from 1 to 500 mM sodium phosphate, pH 7.3, in 50 mM increments. The protein content of the eluted fractions was determined by Bradford assay [27], concentrated and subjected to SDS-PAGE analysis. The fraction containing the porin trimer was treated with 1% octyl-POE and 2.5% SDS at 100°C to monitor the dissociation of the trimer into monomers (Fig. S2).

### SDS-PAGE Analysis

Gel electrophoresis was performed using 9% SDS polyacrylamide gels in a BioRad Mini-PROTEAN® II Electrophoresis Cell. Loading buffer was composed of 0.5 M Tris pH 6.8, 10% glycerol, 10% SDS, 5% 2-mercaptoethanol and 1% bromophenol blue. A 10 µL amount of loading buffer was mixed with 30 µL of sample and boiled for 5 min. Following electrophoresis for 4 h at room temperature, gels were fixed and stained with 0.1% Coomassie Brilliant Blue overnight and then destained with an acetic acid/water/methanol mixture (10/40/50). Gels were then rinsed with deionized water and photographed under white light. The extracted gel bands were tryptically digested for LC-MS/MS analysis as previously described [28].

### Capillary LC-MS/MS Analysis

Proteomic analysis was used to identify the sequences of the proteins in the toga fraction. Identification of proteins was achieved through the detection of unique peptides with LC/MS/MS. Results were compared to a single organism database containing the *T. maritima* genome sequence.

The capillary RPLC system used for peptide separations has been previously described [29]. Briefly, the HPLC system consisted of a custom configuration of 100-mL ISCO Model 100 DM syringe pumps (Isco, Inc., Lincoln, NE), 2-position Valco valves (Valco Instruments Co., Houston, TX), and a PAL autosampler (Leap Technologies, Carrboro, NC), allowing for fully automated sample analysis across four separate HPLC columns (3-µm Jupiter C_18_ stationary phase, Phenomenex, Torrence, CA). Mobile phase consisted of 0.1% formic acid in water (A) and 0.1% formic acid acetonitrile (B). The HPLC system was equilibrated at 10 kpsi with 100% mobile phase A, and a mobile phase selection valve was switched 50 min after injection, which created a near-exponential gradient as mobile phase B displaced A in a 2.5 mL active mixer. A 40-cm length of 360 µm o.d. x 15 µm i.d. fused silica tubing was used to split ∼17 µL/min of flow before it reached the injection valve (5 µL sample loop). The split flow controlled the gradient speed under conditions of constant pressure operation (10 kpsi). Flow through the capillary HPLC column when equilibrated to 100% mobile phase A was ∼500 nL/min. ESI using an etched fused-silica tip [29] was employed to interface the RPLC separation to a LTQ Orbitrap Velos mass spectrometer (Thermo Scientific, San Jose, CA). Precursor ion mass spectra (AGC 1×10^6^) were collected for 400–2000 *m*/*z* range at a resolution of 100 K followed by data dependent ion trap CID MS/MS (collision energy 35%, AGC 3×10^4^) of the ten most abundant ions. A dynamic exclusion time of 180 sec was used to discriminate against previously analyzed ions.

MS/MS data were processed using SEQUEST [30] and a database that contained the genome-derived possible *T. maritima* protein sequences. No enzyme rules were applied, and identified peptides were filtered using the MSGF [31] score of 1E-10 as a cutoff value for confident identifications.

### Circular Dichroism

Circular dichroism experiments were performed on an Applied Photophysics π*-180 instrument with temperature maintained at 37°C. Scans were collected from 200 to 250 nm on a 300 µl sample in a 1 mm path-length quartz cuvette. Samples contained 0.3 mg/ml protein in 20 mM sodium phosphate buffer, pH 7.3.

### Computational Analyses

Genome and sequences were obtained from the NCBI database. Molecular weight and amino acid composition was calculated using ProtParam [32]. Signal peptides were detected using SignalP, PSort and SecretoryP. Non-classically secreted proteins were predicted by sequence analysis with SecretomeP [33]. Pfam and InterProScan were used to examine the sequences for domains [34], [35]. Beta sheet content analysis of amino acid sequences was carried out using STRAP and SCRATCH [36], [37]. Globularity was predicted using the program GlobPlot2 [38]. Protein hydrophobicity was examined using the Kyte-Doolittle scale implemented at Molecular Toolkit. Alpha helical content was assessed using NetsurfP and Predictprotein. Two dimensional representations of proteins were generated using PredTMBB [39].

### Phylogenetic Analyses

For phylogenetic analyses, protein sequences were obtained from GenBank and potential homologs assembled using the predicted amino acid sequences of OmpA1 and OmpB as BLASTp queries to the GenBank nonredundant database. OmpB homologs were also determined using PSI-BLAST [40]. TM0476 was used as a query sequence and 5 iterations were run using an E-value cut off of 1E-6. Sequences were aligned using MUSCLE [41] as implemented in Seaview [42]. Operons were determined using the ProOpDB database [15]. ProtTest version 2.4 [43] was used to determine the most appropriate substitution model from the ones implemented in the different programs. The maximum likelihood phylogeny, approximate Likelihood Ratio Test (aLRT), and bootstrap support values were determined using PHYML v3.0 [44] with an additional five random starting trees, the Nearest Neighbor Interchange plus Subtree Pruning and Regrafting tree search options, the LG substitution model [45], and a Gamma distribution plus invariant sites to describe Among Site Rate Variation (ASRV). The shape parameter and the percent invariant sites were optimized. Bipartitions in the maximum likelihood tree that had aLRT support values below 0.75 were collapsed and branch lengths were recalculated in TREEPUZZLE using the usertree option, the WAG substitution model [46], and modeling ASRV as described above. Posterior probabilities were calculated using MrBayes 3.1.2 using the WAG substitution model, and a gamma distribution with variable shape parameter. After 1 million generations, the average standard deviation of split frequencies was 0.004031. The first 4000 generations were discarded as burnin.

## Supporting Information

Figure S1 **Preparative SDS-PAGE of a sucrose gradient fraction with the nine bands that were excised and analyzed by LC/MS/MS indicated.** An ultracentrifugation pellet fraction of cell free extract was resolved on a sucrose gradient and fraction 18 from within the 50–65% sucrose fraction was mildly denatured by boiling for 5 min in 2.5% SDS prior to SDS-PAGE. The resulting gel band is shown here. Nine bands were excised and subjected to proteomic analysis by LC/MS/MS. The proteins identified from each band are listed in Table S1.
(TIFF)

Figure S2 **Preparative SDS-PAGE of eight hydroxyapatite fractions.** The proteins in the 50–65% sucrose fractions (fractions 15–21) of a sucrose gradient were pooled and resolved by hydroxyapatite chromatography into eight fractions as described in Materials and Methods. SDS-PAGE analyses of the resulting fractions are shown. A. Proteins from each hydroxyapatite fraction were loaded following mild denaturation (2.5% SDS, 100°C 5 min). B. Proteins from the same eight fractions as in A loaded following complete denaturation (2.5% SDS, 1% octyl-POE, 100°C, 10 min). Asterisks next to bands at the bottom of lanes 6′ and 7′ indicate bands removed for LC/MS/MS (Table S2). Note that A and B show different SDS gels.
(TIFF)

Figure S3 **Circular dichroism spectra of putative OmpB.** Scans were collected from 200 to 250 nm at 37°C with protein concentrations of 0.3 mg/ml protein. The purified porin, displayed predominantly β-like characteristics.
(TIF)

Table S1 **Full catalogue of proteins in the bands of the SDS-PAGE gel of fraction 18 of the sucrose gradient as shown in Figure S1**. Proteins in bands 1–9 were identified by two or more unique peptide matches to a database that contained the genome-derived possible *T. maritima* protein sequences. OmpA1 (TM0477) and OmpB (TM0476) are highlighted in bold font.
(DOCX)

Table S2 **Catalogue of proteins detected in the fully denatured final fractions of the hydroxyapatite column, lanes 6′ and 7′, Figure S2.** Proteins were identified by 2 or more unique peptide matches. OmpA1 (TM0477) and OmpB (TM0476) are highlighted in bold font.
(DOCX)
