# Supplementary figures and images for: Genes for the Major Structural Components of Thermotogales Species’ Togas Revealed by Proteomic and Evolutionary Analyses of OmpA and OmpB Homologs
Source: PLoS One. 2012 Jun 29;7(6):e40236. doi: 10.1371/journal.pone.0040236 (PMC3387000; doi:10.1371/journal.pone.0040236)

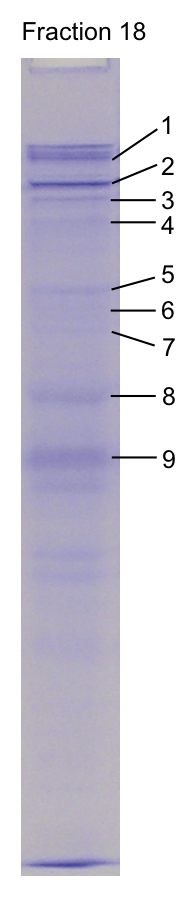

Supplement: Figure S1 — Preparative SDS-PAGE of a sucrose gradient fraction with the nine bands that were excised and analyzed by LC/MS/MS indicated. An ultracentrifugation pellet fraction of cell free extract was resolved on a sucrose gradient and fraction 18 from within the 50–65% sucrose fraction was mildly denatured by boiling for 5 min in 2.5% SDS prior to SDS-PAGE. The resulting gel band is shown here. Nine bands were excised and subjected to proteomic analysis by LC/MS/MS. The proteins identified from each band are listed in Table S1. (TIFF) [file pone.0040236.s001.tiff]

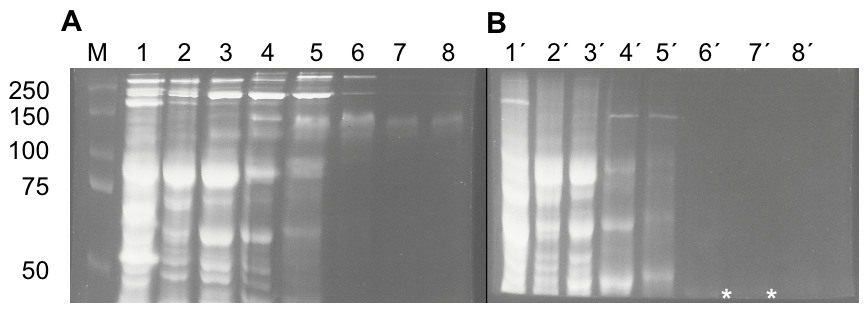

Supplement: Figure S2 — Preparative SDS-PAGE of eight hydroxyapatite fractions. The proteins in the 50–65% sucrose fractions (fractions 15–21) of a sucrose gradient were pooled and resolved by hydroxyapatite chromatography into eight fractions as described in Materials and Methods. SDS-PAGE analyses of the resulting fractions are shown. A. Proteins from each hydroxyapatite fraction were loaded following mild denaturation (2.5% SDS, 100°C 5 min). B. Proteins from the same eight fractions as in A loaded following complete denaturation (2.5% SDS, 1% octyl-POE, 100°C, 10 min). Asterisks next to bands at the bottom of lanes 6′ and 7′ indicate bands removed for LC/MS/MS (Table S2). Note that A and B show different SDS gels. (TIFF) [file pone.0040236.s002.tiff]

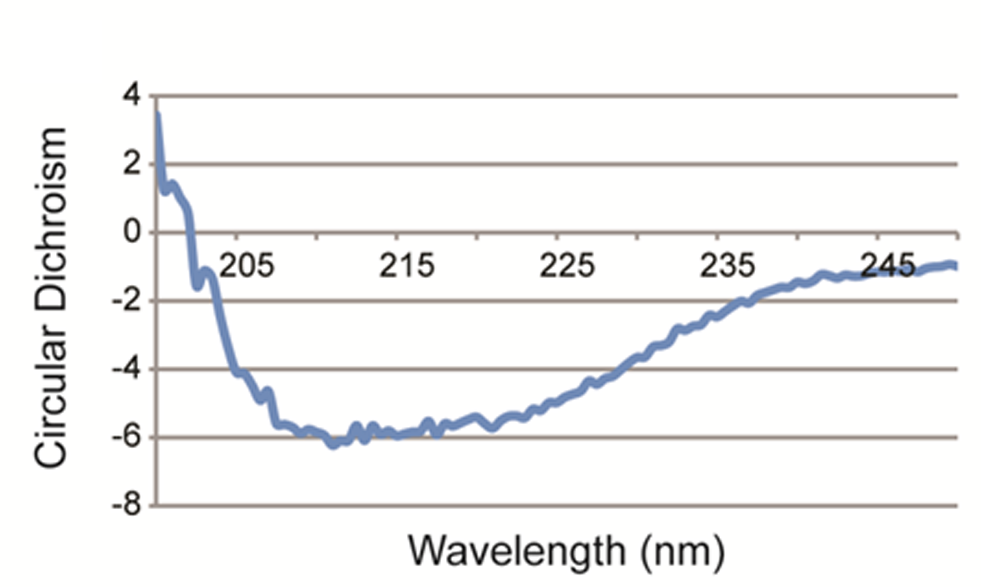

Supplement: Figure S3 — Circular dichroism spectra of putative OmpB. Scans were collected from 200 to 250 nm at 37°C with protein concentrations of 0.3 mg/ml protein. The purified porin, displayed predominantly β-like characteristics. (TIF) [file pone.0040236.s003.tif]
