# Supplementary material for: Genes for the Major Structural Components of Thermotogales Species’ Togas Revealed by Proteomic and Evolutionary Analyses of OmpA and OmpB Homologs
Source: PLoS One. 2012 Jun 29;7(6):e40236. doi: 10.1371/journal.pone.0040236 (PMC3387000; doi:10.1371/journal.pone.0040236)
Supplement: Table S1 — Full catalogue of proteins in the bands of the SDS-PAGE gel of fraction 18 of the sucrose gradient as shown in Figure S1. Proteins in bands 1–9 were identified by two or more unique peptide matches to a database that contained the genome-derived possible T. maritima protein sequences. OmpA1 (TM0477) and OmpB (TM0476) are highlighted in bold font. (DOCX) [file pone.0040236.s004.docx]

**Supplemental Table S-1. Full catalogue of proteins in the bands of the SDS-PAGE gel of fraction 18 of the sucrose gradient as shown in Supplemental Fig. S-1**. Proteins in bands 1-9 were identified by two or more unique peptide matches to a database that contained the genome-derived possible T. maritima protein sequences. OmpA1 (TM0477) and OmpB (TM0476) are highlighted in bold font.

| **gi #** | **Locus** | **Protein Identity** | **Hits** |
| --- | --- | --- | --- |
| **Band 1** | | | |
| 15642792 | TM0017 | pyruvate ferredoxin oxidoreductase, alpha subunit | 8 |
| 15642793 | TM0018 | pyruvate ferredoxin oxidoreductase, beta subunit | 2 |
| 15642800 | TM0025 | beta-glucosidase | 39 |
| 15642806 | TM0031 | oligopeptide ABC transporter, periplasmic oligopeptide-binding protein | 7 |
| 15642807 | TM0032 | transcriptional regulator, XylR-related | 3 |
| 15642809 | TM0034 | iron-sulfur cluster-binding protein | 2 |
| 15642828 | TM0053 | esterase, putative | 2 |
| 15642851 | TM0076 | xylosidase | 8 |
| 15642863 | TM0088 | comE protein, putative | 8 |
| 15642886 | TM0111 | alcohol dehydrogenase, iron-containing | 3 |
| 15642893 | TM0118 | ribonucleotide reductase, B12-dependent | 5 |
| 15642927 | TM0153 | hypothetical protein | 3 |
| 15642947 | TM0173 | reverse gyrase | 4 |
| 15642969 | TM0196 | hypothetical protein | 3 |
| 15642981 | TM0208 | pyruvate kinase | 4 |
| 15643042 | TM0272 | pyruvate phosphate dikinase | 32 |
| 15643058 | TM0289 | 6-phosphofructokinase, pyrophosphate-dependent | 2 |
| 15643077 | TM0308 | alpha-xylosidase YicI | 17 |
| 15643082 | TM0313 | K+ channel, beta subunit | 3 |
| 15643135 | TM0367 | hypothetical protein | 2 |
| 15643146 | TM0379 | NADH oxidase | 4 |
| 15643152 | TM0386 | bacterioferritin comigratory protein/NADH dehydrogenase | 9 |
| 15643160 | TM0394 | hypothetical protein | 7 |
| 15643163 | TM0397 | glutamate synthase, alpha subunit | 32 |
| 15643164 | TM0398 | hypothetical protein | 12 |
| 15643189 | TM0423 | glycerol dehydrogenase | 3 |
| 15643224 | TM0458 | DNA-directed RNA polymerase subunit beta | 68 |
| 15643225 | TM0459 | DNA-directed RNA polymerase, beta' subunit | 89 |
| 15643239 | TM0473 | pyridoxal biosynthesis lyase PdxS | 2 |
| 15643240 | TM0474 | hypothetical protein | 4 |
| **15643242** | **TM0476** | **hypothetical protein (putative OmpB)** | **12** |
| **15643243** | **TM0477** | **outer membrane protein alpha (OmpA1)** | **35** |
| 15643272 | TM0506 | chaperonin GroEL | 27 |
| 15643313 | TM0547 | aspartokinase II | 2 |
| 15643314 | TM0548 | acetolactate synthase, large subunit | 2 |
| 15643316 | TM0550 | ketol-acid reductoisomerase | 7 |
| 15643319 | TM0553 | 2-isopropylmalate synthase | 3 |
| 15643323 | TM0557 | carbamoyl-phosphate synthase, large subunit | 5 |
| 15643337 | TM0571 | heat shock serine protease, periplasmic | 9 |
| 15643403 | TM0638 | polysaccharide export protein, putative | 29 |
| 15643407 | TM0642 | hypothetical protein | 2 |
| 15643419 | TM0654 | spermidine synthase | 2 |
| 15643451 | TM0688 | glyceraldehyde-3-phosphate dehydrogenase | 12 |
| 15643452 | TM0689 | bifunctional phosphoglycerate kinase/triosephosphate isomerase | 47 |
| 15643465 | TM0702 | chemotaxis sensor histidine kinase CheA | 26 |
| 15643479 | TM0716 | propionyl-CoA carboxylase, beta subunit | 6 |
| 15643485 | TM0722 | vacB protein | 5 |
| 15643488 | TM0725 | hypothetical protein | 3 |
| 15643530 | TM0767 | maltodextrin glycosyltransferase | 8 |
| 15643548 | TM0785 | bacteriocin | 16 |
| 15643550 | TM0787 | ribulose-1,5-biphosphate synthetase | 8 |
| 15643585 | TM0822 | phenylalanyl-tRNA synthetase subunit beta | 25 |
| 15643604 | TM0841 | S-layer-like array protein | 2 |
| 15643629 | TM0866 | hypothetical protein | 3 |
| 15643639 | TM0877 | phosphopyruvate hydratase | 24 |
| 15643705 | TM0943 | glutamine synthetase | 15 |
| 15643713 | TM0951 | hypothetical protein | 6 |
| 15643721 | TM0961 | hypothetical protein | 5 |
| 15643722 | TM0962 | hypothetical protein | 3 |
| 15643724 | TM0964 | hypothetical protein | 2 |
| 15643747 | TM0987 | hypothetical protein | 2 |
| 15643772 | TM1014 | hypothetical protein | 2 |
| 15643843 | TM1085 | methionyl-tRNA synthetase | 5 |
| 15643852 | TM1095 | adenylosuccinate lyase | 2 |
| 15643885 | TM1128 | ferritin | 4 |
| 15643905 | TM1148 | isocitrate dehydrogenase | 2 |
| 15643912 | TM1155 | glucose-6-phosphate 1-dehydrogenase | 5 |
| 15643949 | TM1193 | beta-galactosidase | 34 |
| 15643951 | TM1195 | beta-galactosidase | 14 |
| 15644013 | TM1257 | hypothetical protein | 8 |
| 15644028 | TM1272 | aspartyl/glutamyl-tRNA amidotransferase subunit A | 2 |
| 15644030 | TM1274 | hypothetical protein | 4 |
| 15644032 | TM1276 | sugar ABC transporter, ATP-binding protein | 4 |
| 15644097 | TM1345 | polynucleotide phosphorylase/polyadenylase | 9 |
| 15644099 | TM1347 | inosine-5'-monophosphate dehydrogenase | 4 |
| 15644111 | TM1359 | sensor histidine kinase | 5 |
| 15644113 | TM1361 | isoleucyl-tRNA synthetase | 12 |
| 15644176 | TM1425 | Fe-hydrogenase, subunit beta | 2 |
| 15644177 | TM1426 | Fe-hydrogenase, subunit alpha | 5 |
| 15644180 | TM1429 | glycerol uptake facilitator protein | 2 |
| 15644184 | TM1433 | oxidoreductase, putative | 3 |
| 15644221 | TM1472 | DNA-directed RNA polymerase subunit alpha | 4 |
| 15644245 | TM1497 | 50S ribosomal protein L2 | 3 |
| 15644250 | TM1502 | elongation factor Tu | 5 |
| 15644287 | TM1539 | flagellar basal body P-ring protein | 2 |
| 15644360 | TM1612 | F0F1 ATP synthase subunit alpha | 2 |
| 15644363 | TM1615 | F0F1 ATP synthase subunit C | 4 |
| 15644372 | TM1624 | beta-mannosidase, putative | 2 |
| 15644435 | TM1687 | DNA/pantothenate metabolism flavoprotein | 26 |
| 15644439 | TM1691 | hypothetical protein | 4 |
| 15644452 | TM1704 | hypothetical protein | 3 |
| 15644475 | TM1729 | outer membrane protein | 3 |
| 15644525 | TM1780 | argininosuccinate synthase | 4 |
| 15644545 | TM1801 | hypothetical protein | 2 |
| 15644561 | TM1817 | valyl-tRNA synthetase | 5 |
| 15644566 | TM1822 | ftsH protease activity modulator HflK | 3 |
| 15644567 | TM1823 | ftsH protease activity modulator HflC | 4 |
| 15644579 | TM1835 | cyclomaltodextrinase, putative | 3 |
| 15644580 | TM1836 | maltose ABC transporter, permease protein | 3 |
| 15644582 | TM1839 | maltose ABC transporter, periplasmic maltose-binding protein | 7 |
| 15644584 | TM1841 | hypothetical protein | 2 |
| 15644586 | TM1843 | hypothetical protein | 5 |
| 15644587 | TM1844 | hypothetical protein | 10 |
| 15644621 | TM1878 | bifunctional UDP-sugar hydrolase/5'-nucleotidase periplasmic precursor | 4 |
| **Band 2** | | | |
| 15642792 | TM0017 | pyruvate ferredoxin oxidoreductase, alpha subunit | 3 |
| 15642800 | TM0025 | beta-glucosidase | 10 |
| 15642851 | TM0076 | xylosidase | 3 |
| 15642981 | TM0208 | pyruvate kinase | 2 |
| 15643042 | TM0272 | pyruvate phosphate dikinase | 8 |
| 15643058 | TM0289 | 6-phosphofructokinase, pyrophosphate-dependent | 3 |
| 15643077 | TM0308 | alpha-xylosidase YicI | 12 |
| 15643152 | TM0386 | bacterioferritin comigratory protein/NADH dehydrogenase | 5 |
| 15643163 | TM0397 | glutamate synthase, alpha subunit | 11 |
| 15643224 | TM0458 | DNA-directed RNA polymerase subunit beta | 34 |
| 15643225 | TM0459 | DNA-directed RNA polymerase, beta' subunit | 43 |
| 15643239 | TM0473 | pyridoxal biosynthesis lyase PdxS | 2 |
| **15643242** | **TM0476** | **hypothetical protein (putative OmpB)** | **12** |
| **15643243** | **TM0477** | **OMP alpha (OmpA1)** | **9** |
| 15643272 | TM0506 | chaperonin GroEL | 12 |
| 15643316 | TM0550 | ketol-acid reductoisomerase | 6 |
| 15643403 | TM0638 | polysaccharide export protein, putative | 18 |
| 15643451 | TM0688 | glyceraldehyde-3-phosphate dehydrogenase | 3 |
| 15643452 | TM0689 | bifunctional phosphoglycerate kinase/triosephosphate isomerase | 12 |
| 15643465 | TM0701 | chemotaxis sensor histidine kinase CheA | 7 |
| 15643479 | TM0716 | propionyl-CoA carboxylase, beta subunit | 3 |
| 15643530 | TM0767 | maltodextrin glycosyltransferase | 6 |
| 15643548 | TM0785 | bacteriocin | 4 |
| 15643550 | TM0787 | ribulose-1,5-biphosphate synthetase | 5 |
| 15643585 | TM0822 | phenylalanyl-tRNA synthetase subunit beta | 13 |
| 15643639 | TM0877 | phosphopyruvate hydratase | 23 |
| 15643684 | TM0922 | hypothetical protein | 2 |
| 15643705 | TM0944 | glutamine synthetase | 33 |
| 15643713 | TM0951 | hypothetical protein | 3 |
| 15643885 | TM1128 | ferritin | 4 |
| 15644097 | TM1345 | polynucleotide phosphorylase/polyadenylase | 2 |
| 15644113 | TM1361 | isoleucyl-tRNA synthetase | 4 |
| 15644372 | TM1624 | beta-mannosidase, putative | 2 |
| 15644475 | TM1729 | outer membrane protein | 2 |
| 15644587 | TM1844 | hypothetical protein | 4 |
| 15643337 | TM571 | heat shock serine protease, periplasmic | 9 |
| **Band 3** | | | |
| 15642806 | TM0031 | oligopeptide ABC transporter, periplasmic oligopeptide-binding protein | 2 |
| 15642851 | TM0076 | xylosidase | 2 |
| 15643042 | TM0272 | pyruvate phosphate dikinase | 10 |
| 15643077 | TM0308 | alpha-xylosidase YicI | 11 |
| 15643133 | TM0365 | putative aminopeptidase 1 | 2 |
| 15643141 | TM0373 | molecular chaperone DnaK | 2 |
| 15643152 | TM0386 | bacterioferritin comigratory protein/NADH dehydrogenase | 3 |
| 15643163 | TM0397 | glutamate synthase, alpha subunit | 5 |
| 15643224 | TM0458 | DNA-directed RNA polymerase subunit beta | 36 |
| 15643225 | TM0459 | DNA-directed RNA polymerase, beta' subunit | 35 |
| 15643239 | TM0473 | pyridoxal biosynthesis lyase PdxS | 2 |
| **15643242** | **TM0476** | **hypothetical protein (putative OmpB)** | **9** |
| **15643243** | **TM0477** | **Omp alpha (OmpA1)** | **6** |
| 15643272 | TM0506 | chaperonin GroEL | 4 |
| 15643316 | TM0550 | ketol-acid reductoisomerase | 23 |
| 15643337 | TM0571 | heat shock serine protease, periplasmic | 33 |
| 15643403 | TM0638 | polysaccharide export protein, putative | 19 |
| 15643452 | TM0689 | bifunctional phosphoglycerate kinase/triosephosphate isomerase | 7 |
| 15643465 | TM0702 | chemotaxis sensor histidine kinase CheA | 15 |
| 15643479 | TM0716 | propionyl-CoA carboxylase, beta subunit | 2 |
| 15643530 | TM0767 | maltodextrin glycosyltransferase | 3 |
| 15643550 | TM0787 | ribulose-1,5-biphosphate synthetase | 3 |
| 15643585 | TM0822 | phenylalanyl-tRNA synthetase subunit beta | 16 |
| 15643639 | TM0877 | phosphopyruvate hydratase | 15 |
| 15643684 | TM0922 | hypothetical protein | 6 |
| 15643705 | TM0943 | glutamine synthetase | 11 |
| 15643885 | TM1128 | ferritin | 7 |
| 15644009 | TM1253 | NAD synthetase | 9 |
| 15644097 | TM1345 | polynucleotide phosphorylase/polyadenylase | 2 |
| 15644245 | TM1496 | 50S ribosomal protein L2 | 2 |
| 15644372 | TM1624 | beta-mannosidase, putative | 21 |
| 15644580 | TM1836 | maltose ABC transporter, permease protein | 2 |
| **Band 4** | | | |
| 15643152 | TM0386 | bacterioferritin comigratory protein/NADH dehydrogenase | 3 |
| 15643272 | TM0506 | chaperonin GroEL | 12 |
| 15643465 | TM0702 | chemotaxis sensor histidine kinase CheA | 14 |
| 15643224 | TM0458 | DNA-directed RNA polymerase subunit beta | 8 |
| 15643225 | TM0459 | DNA-directed RNA polymerase, beta' subunit | 23 |
| 15643885 | TM1128 | ferritin | 13 |
| 15643337 | TM0571 | heat shock serine protease, periplasmic | 10 |
| 15643242 | TM0476 | hypothetical protein | 4 |
| 15643713 | TM0951 | hypothetical protein | 3 |
| 15643316 | TM0550 | ketol-acid reductoisomerase | 8 |
| 15644580 | TM1836 | maltose ABC transporter, permease protein | 3 |
| 15643141 | TM0373 | molecular chaperone DnaK | 2 |
| **15643243** | **TM0477** | **OMP alpha (OmpA1)** | **6** |
| 15643585 | TM0822 | phenylalanyl-tRNA synthetase subunit beta | 11 |
| 15643639 | TM0877 | phosphopyruvate hydratase | 14 |
| 15643403 | TM0638 | polysaccharide export protein, putative | 23 |
| 15643133 | TM0365 | putative aminopeptidase 1 | 9 |
| 15642947 | TM0173 | reverse gyrase | 2 |
| 15643550 | TM0787 | ribulose-1,5-biphosphate synthetase | 2 |
| **Band 5** | | | |
| 15642792 | TM0017 | pyruvate ferredoxin oxidoreductase, alpha subunit | 9 |
| 15642800 | TM0025 | beta-glucosidase | 11 |
| 15642806 | TM0031 | oligopeptide ABC transporter, periplasmic oligopeptide-binding protein | 10 |
| 15642809 | TM0034 | iron-sulfur cluster-binding protein | 3 |
| 15642839 | TM0064 | glucuronate isomerase | 3 |
| 15642851 | TM0076 | xylosidase | 48 |
| 15642863 | TM0088 | comE protein, putative | 4 |
| 15642886 | TM0111 | alcohol dehydrogenase, iron-containing | 4 |
| 15642893 | TM0118 | ribonucleotide reductase, B12-dependent | 2 |
| 15642927 | TM0153 | hypothetical protein | 8 |
| 15642930 | TM0156 | alkaline phosphatase | 3 |
| 15642942 | TM0168 | leucyl-tRNA synthetase | 2 |
| 15642969 | TM0196 | hypothetical protein | 2 |
| 15642981 | TM0208 | pyruvate kinase | 6 |
| 15642990 | TM0217 | glycyl-tRNA synthetase, beta subunit | 6 |
| 15643042 | TM0272 | pyruvate phosphate dikinase | 36 |
| 15643058 | TM0289 | 6-phosphofructokinase, pyrophosphate-dependent | 2 |
| 15643064 | TM0295 | putative translaldolase | 3 |
| 15643082 | TM0313 | K+ channel, beta subunit | 6 |
| 15643146 | TM0379 | NADH oxidase | 7 |
| 15643152 | TM0386 | bacterioferritin comigratory protein/NADH dehydrogenase | 5 |
| 15643189 | TM0423 | glycerol dehydrogenase | 6 |
| 15643195 | TM0429 | methyl-accepting chemotaxis protein | 2 |
| 15643224 | TM0458 | DNA-directed RNA polymerase subunit beta | 46 |
| 15643225 | TM0459 | DNA-directed RNA polymerase, beta' subunit | 62 |
| 15643239 | TM0473 | pyridoxal biosynthesis lyase PdxS | 4 |
| 15643240 | TM0474 | hypothetical protein | 13 |
| **15643242** | **TM0476** | **hypothetical protein (putative OmpB)** | **17** |
| **15643243** | **TM0477** | **outer membrane protein alpha (OmpA1)** | **16** |
| 15643272 | TM0506 | chaperonin GroEL | 35 |
| 15643313 | TM0547 | aspartokinase II | 5 |
| 15643316 | TM0550 | ketol-acid reductoisomerase | 2 |
| 15643318 | TM0552 | putative alpha-isopropylmalate/homocitrate synthase family transferase | 2 |
| 15643319 | TM0553 | 2-isopropylmalate synthase | 4 |
| 15643323 | TM0557 | carbamoyl-phosphate synthase, large subunit | 5 |
| 15643337 | TM0571 | heat shock serine protease, periplasmic | 20 |
| 15643361 | TM0595 | sugar ABC transporter, periplasmic sugar-binding protein, putative | 2 |
| 15643403 | TM0638 | polysaccharide export protein, putative | 42 |
| 15643404 | TM0639 | hypothetical protein | 2 |
| 15643419 | TM0654 | spermidine synthase | 3 |
| 15643451 | TM0688 | glyceraldehyde-3-phosphate dehydrogenase | 6 |
| 15643452 | TM0689 | bifunctional phosphoglycerate kinase/triosephosphate isomerase | 20 |
| 15643465 | TM0702 | chemotaxis sensor histidine kinase CheA | 28 |
| 15643479 | TM0716 | propionyl-CoA carboxylase, beta subunit | 12 |
| 15643485 | TM0722 | vacB protein | 2 |
| 15643488 | TM0725 | hypothetical protein | 2 |
| 15643514 | TM0751 | uridine kinase-related protein | 2 |
| 15643530 | TM0767 | maltodextrin glycosyltransferase | 4 |
| 15643548 | TM0785 | bacteriocin | 2 |
| 15643550 | TM0787 | ribulose-1,5-biphosphate synthetase | 12 |
| 15643569 | TM0806 | hypothetical protein | 17 |
| 15643584 | TM0821 | phenylalanyl-tRNA synthetase subunit alpha | 8 |
| 15643585 | TM0822 | phenylalanyl-tRNA synthetase subunit beta | 45 |
| 15643596 | TM0833 | DNA gyrase, subunit B | 2 |
| 15643604 | TM0841 | S-layer-like array protein | 3 |
| 15643639 | TM0877 | phosphopyruvate hydratase | 28 |
| 15643705 | TM0943 | glutamine synthetase | 3 |
| 15643713 | TM0951 | hypothetical protein | 4 |
| 15643739 | TM0979 | hypothetical protein | 2 |
| 15643744 | TM0984 | hypothetical protein | 2 |
| 15643747 | TM0987 | hypothetical protein | 2 |
| 15643811 | TM1053 | cationic outer membrane protein | 2 |
| 15643843 | TM1085 | methionyl-tRNA synthetase | 6 |
| 15643885 | TM1128 | ferritin | 2 |
| 15643905 | TM1148 | isocitrate dehydrogenase | 2 |
| 15643912 | TM1155 | glucose-6-phosphate 1-dehydrogenase | 3 |
| 15644028 | TM1272 | aspartyl/glutamyl-tRNA amidotransferase subunit A | 3 |
| 15644032 | TM1276 | sugar ABC transporter, ATP-binding protein | 3 |
| 15644097 | TM1345 | polynucleotide phosphorylase/polyadenylase | 3 |
| 15644099 | TM1347 | inosine-5'-monophosphate dehydrogenase | 4 |
| 15644113 | TM1361 | isoleucyl-tRNA synthetase | 5 |
| 15644168 | TM1416 | hypothetical protein | 3 |
| 15644176 | TM1425 | Fe-hydrogenase, subunit beta | 2 |
| 15644177 | TM1426 | Fe-hydrogenase, subunit alpha | 4 |
| 15644258 | TM1510 | hypothetical protein | 2 |
| 15644367 | TM1619 | DNA-directed DNA polymerase I | 3 |
| 15644372 | TM1624 | beta-mannosidase, putative | 7 |
| 15644453 | TM1705 | lysyl-tRNA synthetase | 4 |
| 15644475 | TM1729 | outer membrane protein | 2 |
| 15644507 | TM1762 | transketolase | 2 |
| 15644561 | TM1817 | valyl-tRNA synthetase | 5 |
| 15644579 | TM1835 | cyclomaltodextrinase, putative | 2 |
| 15644580 | TM1836 | maltose ABC transporter, permease protein | 12 |
| 15644582 | TM1839 | maltose ABC transporter, periplasmic maltose-binding protein | 3 |
| 15644612 | TM1869 | ATP-dependent protease LA, putative | 2 |
| 15644621 | TM1878 | bifunctional UDP-sugar hydrolase/5'-nucleotidase periplasmic precursor | 11 |
| **Band 6** | | | |
| 15642792 | TM0017 | pyruvate ferredoxin oxidoreductase, alpha subunit | 4 |
| 15642798 | TM0023 | methyl-accepting chemotaxis protein | 2 |
| 15642800 | TM0025 | beta-glucosidase | 12 |
| 15642806 | TM0031 | oligopeptide ABC transporter, periplasmic oligopeptide-binding protein | 9 |
| 15642809 | TM0034 | iron-sulfur cluster-binding protein | 3 |
| 15642839 | TM0064 | glucuronate isomerase | 5 |
| 15642851 | TM0076 | xylosidase | 19 |
| 15642863 | TM0088 | comE protein, putative | 3 |
| 15642927 | TM0153 | hypothetical protein | 17 |
| 15642930 | TM0156 | alkaline phosphatase | 2 |
| 15642942 | TM0168 | leucyl-tRNA synthetase | 3 |
| 15642969 | TM0196 | hypothetical protein | 2 |
| 15642981 | TM0208 | pyruvate kinase | 5 |
| 15642990 | TM0217 | glycyl-tRNA synthetase, beta subunit | 2 |
| 15643042 | TM0272 | pyruvate phosphate dikinase | 29 |
| 15643058 | TM0289 | 6-phosphofructokinase, pyrophosphate-dependent | 5 |
| 15643062 | TM0293 | gamma-glutamyl phosphate reductase | 2 |
| 15643064 | TM0295 | putative translaldolase | 4 |
| 15643082 | TM0313 | K+ channel, beta subunit | 20 |
| 15643146 | TM0379 | NADH oxidase | 2 |
| 15643152 | TM0386 | bacterioferritin comigratory protein/NADH dehydrogenase | 10 |
| 15643189 | TM0423 | glycerol dehydrogenase | 2 |
| 15643224 | TM0458 | DNA-directed RNA polymerase subunit beta | 51 |
| 15643225 | TM0459 | DNA-directed RNA polymerase, beta' subunit | 14 |
| 15643239 | TM0473 | pyridoxal biosynthesis lyase PdxS | 4 |
| 15643240 | TM0474 | hypothetical protein | 24 |
| **15643242** | **TM0476** | **hypothetical protein (putative OmpB)** | **13** |
| **15643243** | **TM0477** | **outer membrane protein alpha (OmpA1)** | **20** |
| 15643272 | TM0506 | chaperonin GroEL | 39 |
| 15643313 | TM0547 | aspartokinase II | 5 |
| 15643314 | TM0548 | acetolactate synthase, large subunit | 4 |
| 15643318 | TM0552 | putative alpha-isopropylmalate/homocitrate synthase family transferase | 2 |
| 15643319 | TM0553 | 2-isopropylmalate synthase | 3 |
| 15643323 | TM0557 | carbamoyl-phosphate synthase, large subunit | 9 |
| 15643337 | TM0571 | heat shock serine protease, periplasmic | 27 |
| 15643403 | TM0638 | polysaccharide export protein, putative | 38 |
| 15643407 | TM0642 | hypothetical protein | 2 |
| 15643419 | TM0654 | spermidine synthase | 2 |
| 15643451 | TM0688 | glyceraldehyde-3-phosphate dehydrogenase | 8 |
| 15643452 | TM0689 | bifunctional phosphoglycerate kinase/triosephosphate isomerase | 13 |
| 15643465 | TM0702 | chemotaxis sensor histidine kinase CheA | 22 |
| 15643479 | TM0716 | propionyl-CoA carboxylase, beta subunit | 16 |
| 15643483 | TM0720 | serine hydroxymethyltransferase | 2 |
| 15643485 | TM0722 | vacB protein | 7 |
| 15643488 | TM0725 | hypothetical protein | 2 |
| 15643530 | TM0767 | maltodextrin glycosyltransferase | 3 |
| 15643548 | TM0785 | bacteriocin | 3 |
| 15643550 | TM0787 | ribulose-1,5-biphosphate synthetase | 15 |
| 15643569 | TM0806 | hypothetical protein | 7 |
| 15643584 | TM0821 | phenylalanyl-tRNA synthetase subunit alpha | 7 |
| 15643585 | TM0822 | phenylalanyl-tRNA synthetase subunit beta | 41 |
| 15643596 | TM0833 | DNA gyrase, subunit B | 2 |
| 15643604 | TM0841 | S-layer-like array protein | 2 |
| 15643639 | TM0877 | phosphopyruvate hydratase | 65 |
| 15643678 | TM0916 | hypothetical protein | 3 |
| 15643705 | TM0943 | glutamine synthetase | 5 |
| 15643713 | TM0951 | hypothetical protein | 9 |
| 15643773 | TM1015 | glutamate dehydrogenase | 3 |
| 15643842 | TM1084 | DNA gyrase, subunit A | 3 |
| 15643843 | TM1085 | methionyl-tRNA synthetase | 5 |
| 15643905 | TM1148 | isocitrate dehydrogenase | 5 |
| 15643912 | TM1155 | glucose-6-phosphate 1-dehydrogenase | 2 |
| 15643958 | TM1202 | maltose ABC transporter, permease protein | 3 |
| 15644028 | TM1272 | aspartyl/glutamyl-tRNA amidotransferase subunit A | 5 |
| 15644032 | TM1276 | sugar ABC transporter, ATP-binding protein | 4 |
| 15644099 | TM1347 | inosine-5'-monophosphate dehydrogenase | 2 |
| 15644113 | TM1361 | isoleucyl-tRNA synthetase | 4 |
| 15644137 | TM1385 | glucose-6-phosphate isomerase | 3 |
| 15644152 | TM1400 | aspartate aminotransferase, putative | 3 |
| 15644153 | TM1401 | D-3-phosphoglycerate dehydrogenase | 2 |
| 15644168 | TM1416 | hypothetical protein | 2 |
| 15644170 | TM1419 | myo-inositol-1-phosphate synthase-related protein | 4 |
| 15644176 | TM1425 | Fe-hydrogenase, subunit beta | 3 |
| 15644360 | TM1612 | F0F1 ATP synthase subunit alpha | 2 |
| 15644367 | TM1619 | DNA-directed DNA polymerase I | 4 |
| 15644400 | TM1652 | hypothetical protein | 3 |
| 15644453 | TM1705 | lysyl-tRNA synthetase | 4 |
| 15644475 | TM1729 | outer membrane protein | 6 |
| 15644507 | TM1762 | transketolase | 2 |
| 15644525 | TM1780 | argininosuccinate synthase | 2 |
| 15644561 | TM1817 | valyl-tRNA synthetase | 4 |
| 15644579 | TM1835 | cyclomaltodextrinase, putative | 3 |
| 15644580 | TM1836 | maltose ABC transporter, permease protein | 22 |
| 15644582 | TM1839 | maltose ABC transporter, periplasmic maltose-binding protein | 3 |
| 15644612 | TM1869 | ATP-dependent protease LA, putative | 2 |
| 15644621 | TM1878 | bifunctional UDP-sugar hydrolase/5'-nucleotidase periplasmic precursor | 10 |
| **Band 7** | | | |
| 15642792 | TM0017 | pyruvate ferredoxin oxidoreductase, alpha subunit | 13 |
| 15642799 | TM0024 | laminarinase | 2 |
| 15642800 | TM0025 | beta-glucosidase | 12 |
| 15642806 | TM0031 | oligopeptide ABC transporter, periplasmic oligopeptide-binding protein | 14 |
| 15642809 | TM0034 | iron-sulfur cluster-binding protein | 2 |
| 15642839 | TM0064 | glucuronate isomerase | 26 |
| 15642842 | TM0067 | 2-keto-3-deoxygluconate kinase | 2 |
| 15642863 | TM0088 | comE protein, putative | 32 |
| 15642886 | TM0111 | alcohol dehydrogenase, iron-containing | 6 |
| 15642893 | TM0118 | ribonucleotide reductase, B12-dependent | 2 |
| 15642927 | TM0153 | hypothetical protein | 19 |
| 15642942 | TM0168 | leucyl-tRNA synthetase | 2 |
| 15642958 | TM0184 | phosphoglucosamine mutase | 6 |
| 15642981 | TM0208 | pyruvate kinase | 5 |
| 15643042 | TM0272 | pyruvate phosphate dikinase | 47 |
| 15643051 | TM0282 | aldose 1-epimerase | 9 |
| 15643058 | TM0289 | 6-phosphofructokinase, pyrophosphate-dependent | 3 |
| 15643064 | TM0295 | putative translaldolase | 13 |
| 15643082 | TM0313 | K+ channel, beta subunit | 2 |
| 15643146 | TM0379 | NADH oxidase | 2 |
| 15643152 | TM0386 | bacterioferritin comigratory protein/NADH dehydrogenase | 11 |
| 15643189 | TM0423 | glycerol dehydrogenase | 14 |
| 15643224 | TM0458 | DNA-directed RNA polymerase subunit beta | 111 |
| 15643225 | TM0459 | DNA-directed RNA polymerase, beta' subunit | 15 |
| 15643239 | TM0473 | pyridoxal biosynthesis lyase PdxS | 3 |
| 15643240 | TM0474 | hypothetical protein | 5 |
| **15643242** | **TM0476** | **hypothetical protein (putative OmpB)** | **18** |
| **15643243** | **TM0477** | **outer membrane protein alpha (OmpA1)** | **24** |
| 15643272 | TM0506 | chaperonin GroEL | 44 |
| 15643313 | TM0547 | aspartokinase II | 2 |
| 15643314 | TM0548 | acetolactate synthase, large subunit | 2 |
| 15643318 | TM0552 | putative alpha-isopropylmalate/homocitrate synthase family transferase | 2 |
| 15643319 | TM0553 | 2-isopropylmalate synthase | 3 |
| 15643323 | TM0557 | carbamoyl-phosphate synthase, large subunit | 11 |
| 15643337 | TM0571 | heat shock serine protease, periplasmic | 33 |
| 15643349 | TM0583 | lipopolysaccharide biosynthesis protein | 2 |
| 15643361 | TM0595 | sugar ABC transporter, periplasmic sugar-binding protein, putative | 2 |
| 15643403 | TM0638 | polysaccharide export protein, putative | 52 |
| 15643404 | TM0639 | hypothetical protein | 3 |
| 15643409 | TM0644 | hypothetical protein | 2 |
| 15643451 | TM0688 | glyceraldehyde-3-phosphate dehydrogenase | 7 |
| 15643452 | TM0689 | bifunctional phosphoglycerate kinase/triosephosphate isomerase | 23 |
| 15643465 | TM0702 | chemotaxis sensor histidine kinase CheA | 30 |
| 15643479 | TM0716 | propionyl-CoA carboxylase, beta subunit | 29 |
| 15643483 | TM0720 | serine hydroxymethyltransferase | 2 |
| 15643485 | TM0722 | vacB protein | 5 |
| 15643488 | TM0725 | hypothetical protein | 2 |
| 15643530 | TM0767 | maltodextrin glycosyltransferase | 4 |
| 15643548 | TM0785 | bacteriocin | 3 |
| 15643550 | TM0787 | ribulose-1,5-biphosphate synthetase | 10 |
| 15643569 | TM0806 | hypothetical protein | 3 |
| 15643584 | TM0821 | phenylalanyl-tRNA synthetase subunit alpha | 5 |
| 15643585 | TM0822 | phenylalanyl-tRNA synthetase subunit beta | 26 |
| 15643604 | TM0841 | S-layer-like array protein | 3 |
| 15643639 | TM0877 | phosphopyruvate hydratase | 22 |
| 15643644 | TM0882 | O-acetylhomoserine sulfhydrylase | 2 |
| 15643678 | TM0916 | hypothetical protein | 2 |
| 15643705 | TM0943 | glutamine synthetase | 7 |
| 15643713 | TM0951 | hypothetical protein | 5 |
| 15643773 | TM1015 | glutamate dehydrogenase | 9 |
| 15643842 | TM1084 | DNA gyrase, subunit A | 4 |
| 15643843 | TM1085 | methionyl-tRNA synthetase | 6 |
| 15643885 | TM1128 | ferritin | 2 |
| 15643912 | TM1155 | glucose-6-phosphate 1-dehydrogenase | 4 |
| 15643957 | TM1201 | arabinogalactan endo-1,4-beta-galactosidase, putative | 2 |
| 15643958 | TM1202 | maltose ABC transporter, permease protein | 7 |
| 15644032 | TM1276 | sugar ABC transporter, ATP-binding protein | 7 |
| 15644097 | TM1345 | polynucleotide phosphorylase/polyadenylase | 3 |
| 15644099 | TM1347 | inosine-5'-monophosphate dehydrogenase | 7 |
| 15644113 | TM1361 | isoleucyl-tRNA synthetase | 6 |
| 15644131 | TM1379 | seryl-tRNA synthetase | 2 |
| 15644137 | TM1385 | glucose-6-phosphate isomerase | 4 |
| 15644152 | TM1400 | aspartate aminotransferase, putative | 2 |
| 15644176 | TM1425 | Fe-hydrogenase, subunit beta | 2 |
| 15644177 | TM1426 | Fe-hydrogenase, subunit alpha | 3 |
| 15644179 | TM1428 | methyl-accepting chemotaxis protein | 3 |
| 15644221 | TM1472 | DNA-directed RNA polymerase subunit alpha | 4 |
| 15644360 | TM1612 | F0F1 ATP synthase subunit alpha | 2 |
| 15644367 | TM1619 | DNA-directed DNA polymerase I | 3 |
| 15644400 | TM1652 | hypothetical protein | 3 |
| 15644406 | TM1658 | S-adenosylmethionine synthetase | 3 |
| 15644440 | TM1692 | aminotransferase, class V | 5 |
| 15644475 | TM1729 | outer membrane protein | 10 |
| 15644507 | TM1762 | transketolase | 4 |
| 15644525 | TM1780 | argininosuccinate synthase | 2 |
| 15644545 | TM1801 | hypothetical protein | 2 |
| 15644561 | TM1817 | valyl-tRNA synthetase | 7 |
| 15644579 | TM1835 | cyclomaltodextrinase, putative | 2 |
| 15644580 | TM1836 | maltose ABC transporter, permease protein | 33 |
| 15644582 | TM1839 | maltose ABC transporter, periplasmic maltose-binding protein | 4 |
| 15644587 | TM1844 | hypothetical protein | 2 |
| 15644621 | TM1878 | bifunctional UDP-sugar hydrolase/5'-nucleotidase periplasmic precursor | 21 |
| **Band 8** | | | |
| 15642792 | TM0017 | pyruvate ferredoxin oxidoreductase, alpha subunit | 14 |
| 15642798 | TM0023 | methyl-accepting chemotaxis protein | 2 |
| 15642800 | TM0025 | beta-glucosidase | 13 |
| 15642806 | TM0031 | oligopeptide ABC transporter, periplasmic oligopeptide-binding protein | 21 |
| 15642808 | TM0033 | hypothetical protein | 6 |
| 15642844 | TM0069 | mannonate dehydratase | 2 |
| 15642886 | TM0111 | alcohol dehydrogenase, iron-containing | 5 |
| 15642927 | TM0153 | hypothetical protein | 22 |
| 15642942 | TM0168 | leucyl-tRNA synthetase | 2 |
| 15642954 | TM0180 | hypothetical protein | 2 |
| 15642958 | TM0184 | phosphoglucosamine mutase | 9 |
| 15642969 | TM0196 | hypothetical protein | 4 |
| 15642981 | TM0208 | pyruvate kinase | 5 |
| 15643042 | TM0272 | pyruvate phosphate dikinase | 45 |
| 15643058 | TM0289 | 6-phosphofructokinase, pyrophosphate-dependent | 10 |
| 15643122 | TM0354 | hypothetical protein | 3 |
| 15643140 | TM0372 | cation efflux system protein, putative | 2 |
| 15643146 | TM0379 | NADH oxidase | 10 |
| 15643152 | TM0386 | bacterioferritin comigratory protein/NADH dehydrogenase | 7 |
| 15643189 | TM0423 | glycerol dehydrogenase | 3 |
| 15643195 | TM0429 | methyl-accepting chemotaxis protein | 2 |
| 15643224 | TM0458 | DNA-directed RNA polymerase subunit beta | 23 |
| 15643225 | TM0459 | DNA-directed RNA polymerase, beta' subunit | 10 |
| 15643239 | TM0473 | pyridoxal biosynthesis lyase PdxS | 2 |
| 15643240 | TM0474 | hypothetical protein | 2 |
| **15643242** | **TM0476** | **hypothetical protein (putative OmpB)** | **16** |
| **15643243** | **TM0477** | **outer membrane protein alpha (OmpA1)** | **47** |
| 15643272 | TM0506 | chaperonin GroEL | 36 |
| 15643313 | TM0547 | aspartokinase II | 9 |
| 15643314 | TM0548 | acetolactate synthase, large subunit | 3 |
| 15643318 | TM0552 | putative alpha-isopropylmalate/homocitrate synthase family transferase | 4 |
| 15643319 | TM0553 | 2-isopropylmalate synthase | 2 |
| 15643337 | TM0571 | heat shock serine protease, periplasmic | 58 |
| 15643361 | TM0595 | sugar ABC transporter, periplasmic sugar-binding protein, putative | 4 |
| 15643403 | TM0638 | polysaccharide export protein, putative | 97 |
| 15643404 | TM0639 | hypothetical protein | 3 |
| 15643419 | TM0654 | spermidine synthase | 4 |
| 15643451 | TM0688 | glyceraldehyde-3-phosphate dehydrogenase | 2 |
| 15643452 | TM0689 | bifunctional phosphoglycerate kinase/triosephosphate isomerase | 43 |
| 15643465 | TM0702 | chemotaxis sensor histidine kinase CheA | 22 |
| 15643483 | TM0720 | serine hydroxymethyltransferase | 6 |
| 15643485 | TM0722 | vacB protein | 3 |
| 15643522 | TM0759 | acyltransferase, putative | 2 |
| 15643530 | TM0767 | maltodextrin glycosyltransferase | 4 |
| 15643550 | TM0787 | ribulose-1,5-biphosphate synthetase | 15 |
| 15643553 | TM0790 | hypothetical protein | 14 |
| 15643569 | TM0806 | hypothetical protein | 3 |
| 15643570 | TM0807 | peroxiredoxin | 2 |
| 15643584 | TM0821 | phenylalanyl-tRNA synthetase subunit alpha | 3 |
| 15643585 | TM0822 | phenylalanyl-tRNA synthetase subunit beta | 29 |
| 15643596 | TM0833 | DNA gyrase, subunit B | 2 |
| 15643604 | TM0841 | S-layer-like array protein | 4 |
| 15643629 | TM0866 | hypothetical protein | 2 |
| 15643639 | TM0877 | phosphopyruvate hydratase | 13 |
| 15643644 | TM0882 | O-acetylhomoserine sulfhydrylase | 2 |
| 15643678 | TM0916 | hypothetical protein | 2 |
| 15643705 | TM0943 | glutamine synthetase | 13 |
| 15643713 | TM0951 | hypothetical protein | 3 |
| 15643747 | TM0987 | hypothetical protein | 2 |
| 15643771 | TM1013 | hypothetical protein | 5 |
| 15643772 | TM1014 | hypothetical protein | 4 |
| 15643832 | TM1074 | hypothetical protein | 2 |
| 15643842 | TM1084 | DNA gyrase, subunit A | 17 |
| 15643843 | TM1085 | methionyl-tRNA synthetase | 12 |
| 15643852 | TM1095 | adenylosuccinate lyase | 2 |
| 15643885 | TM1128 | ferritin | 3 |
| 15643905 | TM1148 | isocitrate dehydrogenase | 3 |
| 15643912 | TM1155 | glucose-6-phosphate 1-dehydrogenase | 3 |
| 15643957 | TM1201 | arabinogalactan endo-1,4-beta-galactosidase, putative | 26 |
| 15644009 | TM1253 | NAD synthetase | 3 |
| 15644032 | TM1276 | sugar ABC transporter, ATP-binding protein | 10 |
| 15644041 | TM1286 | 5-methyltetrahydropteroyltriglutamate--homocysteine S-methyltransferase | 2 |
| 15644097 | TM1345 | polynucleotide phosphorylase/polyadenylase | 2 |
| 15644099 | TM1347 | inosine-5'-monophosphate dehydrogenase | 5 |
| 15644113 | TM1361 | isoleucyl-tRNA synthetase | 4 |
| 15644137 | TM1385 | glucose-6-phosphate isomerase | 5 |
| 15644152 | TM1400 | aspartate aminotransferase, putative | 6 |
| 15644168 | TM1416 | hypothetical protein | 4 |
| 15644169 | TM1417 | ABC transporter, ATP-binding protein | 3 |
| 15644170 | TM1419 | myo-inositol-1-phosphate synthase-related protein | 4 |
| 15644176 | TM1425 | Fe-hydrogenase, subunit beta | 3 |
| 15644177 | TM1426 | Fe-hydrogenase, subunit alpha | 4 |
| 15644184 | TM1433 | oxidoreductase, putative | 2 |
| 15644221 | TM1472 | DNA-directed RNA polymerase subunit alpha | 4 |
| 15644250 | TM1502 | elongation factor Tu | 2 |
| 15644258 | TM1510 | hypothetical protein | 2 |
| 15644265 | TM1517 | diaminopimelate decarboxylase | 2 |
| 15644360 | TM1612 | F0F1 ATP synthase subunit alpha | 2 |
| 15644367 | TM1619 | DNA-directed DNA polymerase I | 6 |
| 15644400 | TM1652 | hypothetical protein | 4 |
| 15644406 | TM1658 | S-adenosylmethionine synthetase | 5 |
| 15644414 | TM1666 | diaminopimelate aminotransferase | 2 |
| 15644440 | TM1692 | aminotransferase, class V | 3 |
| 15644475 | TM1729 | outer membrane protein | 8 |
| 15644507 | TM1762 | transketolase | 2 |
| 15644511 | TM1766 | formate--tetrahydrofolate ligase | 10 |
| 15644545 | TM1801 | hypothetical protein | 3 |
| 15644561 | TM1817 | valyl-tRNA synthetase | 7 |
| 15644580 | TM1836 | maltose ABC transporter, permease protein | 13 |
| 15644582 | TM1839 | maltose ABC transporter, periplasmic maltose-binding protein | 5 |
| 15644588 | TM1845 | pullulanase | 4 |
| 15644591 | TM1848 | cellobiose-phosphorylase | 3 |
| 15644595 | TM1852 | hypothetical protein | 17 |
| 15644621 | TM1878 | bifunctional UDP-sugar hydrolase/5'-nucleotidase periplasmic precursor | 89 |
| **Band 9** | | | |
| 15642792 | TM0017 | pyruvate ferredoxin oxidoreductase, alpha subunit | 15 |
| 15642800 | TM0025 | beta-glucosidase | 8 |
| 15642806 | TM0031 | oligopeptide ABC transporter, periplasmic oligopeptide-binding protein | 24 |
| 15642808 | TM0033 | hypothetical protein | 9 |
| 15642809 | TM0034 | iron-sulfur cluster-binding protein | 2 |
| 15642836 | TM0061 | endo-1,4-beta-xylanase A | 6 |
| 15642842 | TM0067 | 2-keto-3-deoxygluconate kinase | 2 |
| 15642846 | TM0071 | oligopeptide ABC transporter, periplasmic oligopeptide-binding protein | 2 |
| 15642886 | TM0111 | alcohol dehydrogenase, iron-containing | 3 |
| 15642927 | TM0153 | hypothetical protein | 14 |
| 15642942 | TM0168 | leucyl-tRNA synthetase | 8 |
| 15642958 | TM0184 | phosphoglucosamine mutase | 2 |
| 15642981 | TM0208 | pyruvate kinase | 7 |
| 15643032 | TM0262 | DNA polymerase III, beta subunit | 2 |
| 15643042 | TM0272 | pyruvate phosphate dikinase | 101 |
| 15643058 | TM0289 | 6-phosphofructokinase, pyrophosphate-dependent | 13 |
| 15643117 | TM0349 | 3-dehydroquinate dehydratase | 4 |
| 15643146 | TM0379 | NADH oxidase | 10 |
| 15643152 | TM0386 | bacterioferritin comigratory protein/NADH dehydrogenase | 8 |
| 15643224 | TM0458 | DNA-directed RNA polymerase subunit beta | 5 |
| 15643238 | TM0472 | glutamine amidotransferase subunit PdxT | 2 |
| 15643239 | TM0473 | pyridoxal biosynthesis lyase PdxS | 6 |
| **15643242** | **TM0476** | **hypothetical protein (putative OmpB)** | **4** |
| **15643243** | **TM0477** | **outer membrane protein alpha (OmpA1)** | **11** |
| 15643272 | TM0506 | chaperonin GroEL | 38 |
| 15643313 | TM0547 | aspartokinase II | 13 |
| 15643314 | TM0548 | acetolactate synthase, large subunit | 12 |
| 15643318 | TM0552 | putative alpha-isopropylmalate/homocitrate synthase family transferase | 3 |
| 15643319 | TM0553 | 2-isopropylmalate synthase | 2 |
| 15643337 | TM0571 | heat shock serine protease, periplasmic | 21 |
| 15643361 | TM0595 | sugar ABC transporter, periplasmic sugar-binding protein, putative | 6 |
| 15643395 | TM0630 | nucleotide sugar epimerase, putative | 2 |
| 15643403 | TM0638 | polysaccharide export protein, putative | 20 |
| 15643451 | TM0688 | glyceraldehyde-3-phosphate dehydrogenase | 3 |
| 15643452 | TM0689 | bifunctional phosphoglycerate kinase/triosephosphate isomerase | 44 |
| 15643465 | TM0702 | chemotaxis sensor histidine kinase CheA | 38 |
| 15643485 | TM0722 | vacB protein | 7 |
| 15643530 | TM0767 | maltodextrin glycosyltransferase | 6 |
| 15643550 | TM0787 | ribulose-1,5-biphosphate synthetase | 9 |
| 15643569 | TM0806 | hypothetical protein | 10 |
| 15643585 | TM0822 | phenylalanyl-tRNA synthetase subunit beta | 24 |
| 15643596 | TM0833 | DNA gyrase, subunit B | 2 |
| 15643604 | TM0841 | S-layer-like array protein | 4 |
| 15643639 | TM0877 | phosphopyruvate hydratase | 3 |
| 15643644 | TM0882 | O-acetylhomoserine sulfhydrylase | 4 |
| 15643680 | TM0918 | methyl-accepting chemotaxis protein | 2 |
| 15643705 | TM0943 | glutamine synthetase | 10 |
| 15643713 | TM0951 | hypothetical protein | 2 |
| 15643739 | TM0979 | hypothetical protein | 2 |
| 15643842 | TM1084 | DNA gyrase, subunit A | 40 |
| 15643843 | TM1085 | methionyl-tRNA synthetase | 2 |
| 15643885 | TM1128 | ferritin | 2 |
| 15643905 | TM1148 | isocitrate dehydrogenase | 3 |
| 15643921 | TM1164 | 2-oxoacid ferredoxin oxidoreductase, alpha subunit | 4 |
| 15643951 | TM1195 | beta-galactosidase | 4 |
| 15643957 | TM1201 | arabinogalactan endo-1,4-beta-galactosidase, putative | 20 |
| 15644028 | TM1272 | aspartyl/glutamyl-tRNA amidotransferase subunit A | 3 |
| 15644032 | TM1276 | sugar ABC transporter, ATP-binding protein | 7 |
| 15644047 | TM1292 | iron-sulfur cluster-binding protein, putative | 2 |
| 15644097 | TM1345 | polynucleotide phosphorylase/polyadenylase | 4 |
| 15644099 | TM1347 | inosine-5'-monophosphate dehydrogenase | 2 |
| 15644113 | TM1361 | isoleucyl-tRNA synthetase | 6 |
| 15644131 | TM1379 | seryl-tRNA synthetase | 4 |
| 15644152 | TM1400 | aspartate aminotransferase, putative | 8 |
| 15644168 | TM1416 | hypothetical protein | 4 |
| 15644170 | TM1419 | myo-inositol-1-phosphate synthase-related protein | 8 |
| 15644176 | TM1425 | Fe-hydrogenase, subunit beta | 2 |
| 15644398 | TM1650 | alpha-amylase, putative | 2 |
| 15644400 | TM1652 | hypothetical protein | 7 |
| 15644406 | TM1658 | S-adenosylmethionine synthetase | 2 |
| 15644440 | TM1692 | aminotransferase, class V | 2 |
| 15644475 | TM1729 | outer membrane protein | 3 |
| 15644507 | TM1762 | transketolase | 2 |
| 15644525 | TM1780 | argininosuccinate synthase | 4 |
| 15644561 | TM1817 | valyl-tRNA synthetase | 5 |
| 15644579 | TM1835 | cyclomaltodextrinase, putative | 2 |
| 15644580 | TM1836 | maltose ABC transporter, permease protein | 39 |
| 15644582 | TM1839 | maltose ABC transporter, periplasmic maltose-binding protein | 9 |
| 15644621 | TM1878 | bifunctional UDP-sugar hydrolase/5'-nucleotidase periplasmic precursor | 53 |

**Supplemental Table S-2. Catalogue of proteins detected in the fully denatured final fractions of the hydroxyapatite column, lanes 6´ and 7´, Supplemental Fig. S-2.** Proteins were identified by 2 or more unique peptide matches. OmpA1 (TM0477) and OmpB (TM0476) are highlighted in bold font.

| **gi #** | **Locus** | **Protein Identity** | **Hits** |
| --- | --- | --- | --- |
| **Lane 6´** | | | |
| 15642781 | TM0006 | muconate cycloisomerase | 2 |
| 15642792 | TM0017 | pyruvate ferredoxin oxidoreductase, alpha subunit | 17 |
| 15642793 | TM0018 | pyruvate ferredoxin oxidoreductase, beta subunit | 6 |
| 15642800 | TM0025 | beta-glucosidase | 3 |
| 15642806 | TM0031 | oligopeptide ABC transporter, periplasmic oligopeptide-binding protein | 6 |
| 15642807 | TM0032 | transcriptional regulator, XylR-related | 11 |
| 15642809 | TM0034 | iron-sulfur cluster-binding protein | 2 |
| 15642958 | TM0111 | alcohol dehydrogenase, iron-containing | 3 |
| 15643010 | TM0184 | phosphoglucosamine mutase | 6 |
| 15643036 | TM0238 | hypothetical protein | 3 |
| 15643041 | TM0271 | hypothetical protein | 4 |
| 15643042 | TM0272 | pyruvate phosphate dikinase | 2 |
| 15643043 | TM0273 | fructose-bisphosphate aldolase | 3 |
| 15643051 | TM0282 | aldose 1-epimerase | 3 |
| 15643058 | TM0289 | 6-phosphofructokinase, pyrophosphate-dependent | 5 |
| 15643189 | TM0355 | hypothetical protein | 5 |
| 15643238 | TM0423 | glycerol dehydrogenase | 3 |
| 15643239 | TM0472 | glutamine amidotransferase subunit PdxT | 27 |
| 15643243 | TM0473 | pyridoxal biosynthesis lyase PdxS | 7 |
| **15643272** | **TM0477** | **outer membrane protein alpha (OmpA1)** | **12** |
| 15643315 | TM0506 | chaperonin GroEL | 2 |
| 15643316 | TM0550 | ketol-acid reductoisomerase | 6 |
| 15643337 | TM0571 | heat shock serine protease, periplasmic | 51 |
| 15643361 | TM0595 | sugar ABC transporter, periplasmic sugar-binding protein, putative | 9 |
| 15643393 | TM0628 | hypothetical protein | 2 |
| 15643395 | TM0630 | nucleotide sugar epimerase, putative | 3 |
| 15643451 | TM0668 | pleiotropic regulatory protein | 9 |
| 15643452 | TM0688 | glyceraldehyde-3-phosphate dehydrogenase | 3 |
| 15643465 | TM0701 | purine-binding chemotaxis protein | 5 |
| 15643474 | TM0702 | chemotaxis sensor histidine kinase CheA | 7 |
| 15643489 | TM0711 | hypothetical protein | 8 |
| 15643490 | TM0726 | tldD protein | 2 |
| 15643525 | TM0762 | 30S ribosomal protein S2 | 2 |
| 15643550 | TM0787 | ribulose-1,5-biphosphate synthetase | 6 |
| 15643551 | TM0788 | thiamine biosynthesis protein ThiC | 2 |
| 15643591 | TM0828 | PfkB family sugar kinase | 3 |
| 15643598 | TM0835 | cell division protein FtsA, putative | 2 |
| 15643639 | TM0877 | phosphopyruvate hydratase | 12 |
| 15643658 | TM0896 | galactose-1-phosphate uridylyltransferase, putative | 2 |
| 15643674 | TM0912 | basic membrane protein, putative | 7 |
| 15643711 | TM0923 | hypothetical protein | 6 |
| 15643724 | TM0949 | LacI family transcription regulator | 2 |
| 15643772 | TM1014 | hypothetical protein | 15 |
| 15643887 | TM1130 | phosphate butyryltransferase | 2 |
| 15643960 | TM1140 | hypothetical protein | 2 |
| 15644013 | TM1204 | maltose ABC transporter, periplasmic maltose-binding protein | 3 |
| 15644026 | TM1270 | cystathionine gamma-synthase | 3 |
| 15644032 | TM1272 | aspartyl/glutamyl-tRNA amidotransferase subunit A | 6 |
| 15644037 | TM1276 | sugar ABC transporter, ATP-binding protein | 4 |
| 15644125 | TM1281 | 6-phospho-beta-glucosidase | 3 |
| 15644152 | TM1373 | hypothetical protein | 5 |
| 15644170 | TM1400 | aspartate aminotransferase, putative | 7 |
| 15644183 | TM1419 | myo-inositol-1-phosphate synthase-related protein | 4 |
| 15644184 | TM1432 | hypothetical protein | 6 |
| 15644250 | TM1433 | oxidoreductase, putative | 2 |
| 15644358 | TM1502 | elongation factor Tu | 5 |
| 15644360 | TM1610 | F0F1 ATP synthase subunit beta | 3 |
| 15644361 | TM1612 | F0F1 ATP synthase subunit alpha | 2 |
| 15644443 | TM1695 | hypothetical protein | 4 |
| 15644468 | TM1721 | hypothetical protein | 52 |
| 15644475 | TM1729 | outer membrane protein | 21 |
| 15644529 | TM1785 | acetylornithine aminotransferase | 7 |
| 15644537 | TM1793 | hypothetical protein | 5 |
| 15644545 | TM1801 | hypothetical protein | 4 |
| 15644551 | TM1807 | hypothetical protein | 2 |
| 15644582 | TM1839 | maltose ABC transporter, periplasmic maltose-binding protein | 25 |
| 15644621 | TM1878 | bifunctional UDP-sugar hydrolase/5'-nucleotidase periplasmic precursor | 2 |
| **Lane 7´** | | | |
| 15642781 | TM0006 | muconate cycloisomerase | 3 |
| 15642792 | TM0017 | pyruvate ferredoxin oxidoreductase, alpha subunit | 13 |
| 15642793 | TM0018 | pyruvate ferredoxin oxidoreductase, beta subunit | 9 |
| 15642800 | TM0025 | beta-glucosidase | 3 |
| 15642806 | TM0031 | oligopeptide ABC transporter periplasmic oligopeptide-binding protein | 4 |
| 15642807 | TM0032 | XylR family transcriptional regulator | 2 |
| 15642886 | TM0111 | alcohol dehydrogenase, iron-containing | 2 |
| 15643010 | TM0238 | hypothetical protein | 2 |
| 15643041 | TM0271 | hypothetical protein | 4 |
| 15643042 | TM0272 | pyruvate phosphate dikinase | 3 |
| 15643043 | TM0273 | fructose-bisphosphate aldolase | 3 |
| 15643123 | TM0355 | hypothetical protein | 4 |
| 15643189 | TM0423 | glycerol dehydrogenase | 6 |
| 15643223 | TM0457 | 50S ribosomal protein L7/L12 | 2 |
| 15643238 | TM0472 | glutamine amidotransferase subunit PdxT | 3 |
| 15643239 | TM0473 | pyridoxal biosynthesis lyase PdxS | 16 |
| **15643242** | **TM0476** | **hypothetical protein (putative OmpB)** | **2** |
| 15643272 | TM0506 | chaperonin GroEL | 16 |
| 15643285 | TM0519 | hypothetical protein | 2 |
| 15643316 | TM0550 | ketol-acid reductoisomerase | 6 |
| 15643337 | TM0571 | heat shock serine protease, periplasmic | 38 |
| 15643361 | TM0595 | sugar ABC transporter periplasmic sugar-binding protein | 6 |
| 15643395 | TM0630 | nucleotide sugar epimerase | 2 |
| 15643433 | TM0668 | pleiotropic regulatory protein | 2 |
| 15643434 | TM0669 | hypothetical protein | 2 |
| 15643451 | TM0688 | glyceraldehyde-3-phosphate dehydrogenase | 6 |
| 15643464 | TM0701 | purine-binding chemotaxis protein | 2 |
| 15643465 | TM0702 | chemotaxis sensor histidine kinase CheA | 2 |
| 15643474 | TM0711 | hypothetical protein | 2 |
| 15643481 | TM0718 | purine-binding chemotaxis protein | 3 |
| 15643489 | TM0726 | tldD protein | 5 |
| 15643490 | TM0727 | pmbA-related protein | 2 |
| 15643550 | TM0787 | ribulose-1,5-biphosphate synthetase | 4 |
| 15643639 | TM0877 | phosphopyruvate hydratase | 7 |
| 15643674 | TM0912 | basic membrane protein | 4 |
| 15643685 | TM0923 | hypothetical protein | 2 |
| 15643711 | TM0949 | LacI family transcription regulator | 2 |
| 15643772 | TM1014 | hypothetical protein | 18 |
| 15643885 | TM1128 | ferritin | 2 |
| 15643887 | TM1130 | phosphate butyryltransferase | 3 |
| 15643892 | TM1135 | branched chain amino acid ABC transporter periplasmic amino acid-binding protein | 2 |
| 15643897 | TM1140 | hypothetical protein | 3 |
| 15643936 | TM1180 | hypothetical protein | 2 |
| 15643960 | TM1204 | maltose ABC transporter periplasmic maltose-binding protein | 2 |
| 15644032 | TM1276 | sugar ABC transporter ATP-binding protein | 5 |
| 15644152 | TM1400 | aspartate aminotransferase | 3 |
| 15644153 | TM1401 | D-3-phosphoglycerate dehydrogenase | 2 |
| 15644170 | TM1419 | myo-inositol-1-phosphate synthase-related protein | 3 |
| 15644184 | TM1433 | oxidoreductase | 2 |
| 15644250 | TM1502 | elongation factor Tu | 2 |
| 15644358 | TM1610 | F0F1 ATP synthase subunit beta | 4 |
| 15644361 | TM1613 | F0F1 ATP synthase subunit delta | 2 |
| 15644376 | TM1628 | ribose-phosphate pyrophosphokinase | 2 |
| 15644400 | TM1652 | hypothetical protein | 3 |
| 15644468 | TM1721 | hypothetical protein | 37 |
| 15644475 | TM1729 | outer membrane protein | 15 |
| 15644529 | TM1785 | acetylornithine aminotransferase | 4 |
| 15644545 | TM1801 | hypothetical protein | 3 |
| 15644553 | TM1809 | hypothetical protein | 2 |
| 15644582 | TM1839 | maltose ABC transporter periplasmic maltose-binding protein | 31 |
| 15644621 | TM1878 | bifunctional UDP-sugar hydrolase/5'-nucleotidase periplasmic precursor | 4 |
