# Supplementary material for: Genes for the Major Structural Components of Thermotogales Species’ Togas Revealed by Proteomic and Evolutionary Analyses of OmpA and OmpB Homologs
Source: PLoS One. 2012 Jun 29;7(6):e40236. doi: 10.1371/journal.pone.0040236 (PMC3387000; doi:10.1371/journal.pone.0040236)
Supplement: Table S2 — Catalogue of proteins detected in the fully denatured final fractions of the hydroxyapatite column, lanes 6′ and 7′, Figure S2. Proteins were identified by 2 or more unique peptide matches. OmpA1 (TM0477) and OmpB (TM0476) are highlighted in bold font. (DOCX) [file pone.0040236.s005.docx]

**Supplemental Table S-2. Catalogue of proteins detected in the fully denatured final fractions of the hydroxyapatite column, lanes 6´ and 7´, Supplemental Fig. S-2.** Proteins were identified by 2 or more unique peptide matches. OmpA1 (TM0477) and OmpB (TM0476) are highlighted in bold font.

| **gi #** | **Locus** | **Protein Identity** | **Hits** |
| --- | --- | --- | --- |
| **Lane 6´** | | | |
| 15642781 | TM0006 | muconate cycloisomerase | 2 |
| 15642792 | TM0017 | pyruvate ferredoxin oxidoreductase, alpha subunit | 17 |
| 15642793 | TM0018 | pyruvate ferredoxin oxidoreductase, beta subunit | 6 |
| 15642800 | TM0025 | beta-glucosidase | 3 |
| 15642806 | TM0031 | oligopeptide ABC transporter, periplasmic oligopeptide-binding protein | 6 |
| 15642807 | TM0032 | transcriptional regulator, XylR-related | 11 |
| 15642809 | TM0034 | iron-sulfur cluster-binding protein | 2 |
| 15642958 | TM0111 | alcohol dehydrogenase, iron-containing | 3 |
| 15643010 | TM0184 | phosphoglucosamine mutase | 6 |
| 15643036 | TM0238 | hypothetical protein | 3 |
| 15643041 | TM0271 | hypothetical protein | 4 |
| 15643042 | TM0272 | pyruvate phosphate dikinase | 2 |
| 15643043 | TM0273 | fructose-bisphosphate aldolase | 3 |
| 15643051 | TM0282 | aldose 1-epimerase | 3 |
| 15643058 | TM0289 | 6-phosphofructokinase, pyrophosphate-dependent | 5 |
| 15643189 | TM0355 | hypothetical protein | 5 |
| 15643238 | TM0423 | glycerol dehydrogenase | 3 |
| 15643239 | TM0472 | glutamine amidotransferase subunit PdxT | 27 |
| 15643243 | TM0473 | pyridoxal biosynthesis lyase PdxS | 7 |
| **15643272** | **TM0477** | **outer membrane protein alpha (OmpA1)** | **12** |
| 15643315 | TM0506 | chaperonin GroEL | 2 |
| 15643316 | TM0550 | ketol-acid reductoisomerase | 6 |
| 15643337 | TM0571 | heat shock serine protease, periplasmic | 51 |
| 15643361 | TM0595 | sugar ABC transporter, periplasmic sugar-binding protein, putative | 9 |
| 15643393 | TM0628 | hypothetical protein | 2 |
| 15643395 | TM0630 | nucleotide sugar epimerase, putative | 3 |
| 15643451 | TM0668 | pleiotropic regulatory protein | 9 |
| 15643452 | TM0688 | glyceraldehyde-3-phosphate dehydrogenase | 3 |
| 15643465 | TM0701 | purine-binding chemotaxis protein | 5 |
| 15643474 | TM0702 | chemotaxis sensor histidine kinase CheA | 7 |
| 15643489 | TM0711 | hypothetical protein | 8 |
| 15643490 | TM0726 | tldD protein | 2 |
| 15643525 | TM0762 | 30S ribosomal protein S2 | 2 |
| 15643550 | TM0787 | ribulose-1,5-biphosphate synthetase | 6 |
| 15643551 | TM0788 | thiamine biosynthesis protein ThiC | 2 |
| 15643591 | TM0828 | PfkB family sugar kinase | 3 |
| 15643598 | TM0835 | cell division protein FtsA, putative | 2 |
| 15643639 | TM0877 | phosphopyruvate hydratase | 12 |
| 15643658 | TM0896 | galactose-1-phosphate uridylyltransferase, putative | 2 |
| 15643674 | TM0912 | basic membrane protein, putative | 7 |
| 15643711 | TM0923 | hypothetical protein | 6 |
| 15643724 | TM0949 | LacI family transcription regulator | 2 |
| 15643772 | TM1014 | hypothetical protein | 15 |
| 15643887 | TM1130 | phosphate butyryltransferase | 2 |
| 15643960 | TM1140 | hypothetical protein | 2 |
| 15644013 | TM1204 | maltose ABC transporter, periplasmic maltose-binding protein | 3 |
| 15644026 | TM1270 | cystathionine gamma-synthase | 3 |
| 15644032 | TM1272 | aspartyl/glutamyl-tRNA amidotransferase subunit A | 6 |
| 15644037 | TM1276 | sugar ABC transporter, ATP-binding protein | 4 |
| 15644125 | TM1281 | 6-phospho-beta-glucosidase | 3 |
| 15644152 | TM1373 | hypothetical protein | 5 |
| 15644170 | TM1400 | aspartate aminotransferase, putative | 7 |
| 15644183 | TM1419 | myo-inositol-1-phosphate synthase-related protein | 4 |
| 15644184 | TM1432 | hypothetical protein | 6 |
| 15644250 | TM1433 | oxidoreductase, putative | 2 |
| 15644358 | TM1502 | elongation factor Tu | 5 |
| 15644360 | TM1610 | F0F1 ATP synthase subunit beta | 3 |
| 15644361 | TM1612 | F0F1 ATP synthase subunit alpha | 2 |
| 15644443 | TM1695 | hypothetical protein | 4 |
| 15644468 | TM1721 | hypothetical protein | 52 |
| 15644475 | TM1729 | outer membrane protein | 21 |
| 15644529 | TM1785 | acetylornithine aminotransferase | 7 |
| 15644537 | TM1793 | hypothetical protein | 5 |
| 15644545 | TM1801 | hypothetical protein | 4 |
| 15644551 | TM1807 | hypothetical protein | 2 |
| 15644582 | TM1839 | maltose ABC transporter, periplasmic maltose-binding protein | 25 |
| 15644621 | TM1878 | bifunctional UDP-sugar hydrolase/5'-nucleotidase periplasmic precursor | 2 |
| **Lane 7´** | | | |
| 15642781 | TM0006 | muconate cycloisomerase | 3 |
| 15642792 | TM0017 | pyruvate ferredoxin oxidoreductase, alpha subunit | 13 |
| 15642793 | TM0018 | pyruvate ferredoxin oxidoreductase, beta subunit | 9 |
| 15642800 | TM0025 | beta-glucosidase | 3 |
| 15642806 | TM0031 | oligopeptide ABC transporter periplasmic oligopeptide-binding protein | 4 |
| 15642807 | TM0032 | XylR family transcriptional regulator | 2 |
| 15642886 | TM0111 | alcohol dehydrogenase, iron-containing | 2 |
| 15643010 | TM0238 | hypothetical protein | 2 |
| 15643041 | TM0271 | hypothetical protein | 4 |
| 15643042 | TM0272 | pyruvate phosphate dikinase | 3 |
| 15643043 | TM0273 | fructose-bisphosphate aldolase | 3 |
| 15643123 | TM0355 | hypothetical protein | 4 |
| 15643189 | TM0423 | glycerol dehydrogenase | 6 |
| 15643223 | TM0457 | 50S ribosomal protein L7/L12 | 2 |
| 15643238 | TM0472 | glutamine amidotransferase subunit PdxT | 3 |
| 15643239 | TM0473 | pyridoxal biosynthesis lyase PdxS | 16 |
| **15643242** | **TM0476** | **hypothetical protein (putative OmpB)** | **2** |
| 15643272 | TM0506 | chaperonin GroEL | 16 |
| 15643285 | TM0519 | hypothetical protein | 2 |
| 15643316 | TM0550 | ketol-acid reductoisomerase | 6 |
| 15643337 | TM0571 | heat shock serine protease, periplasmic | 38 |
| 15643361 | TM0595 | sugar ABC transporter periplasmic sugar-binding protein | 6 |
| 15643395 | TM0630 | nucleotide sugar epimerase | 2 |
| 15643433 | TM0668 | pleiotropic regulatory protein | 2 |
| 15643434 | TM0669 | hypothetical protein | 2 |
| 15643451 | TM0688 | glyceraldehyde-3-phosphate dehydrogenase | 6 |
| 15643464 | TM0701 | purine-binding chemotaxis protein | 2 |
| 15643465 | TM0702 | chemotaxis sensor histidine kinase CheA | 2 |
| 15643474 | TM0711 | hypothetical protein | 2 |
| 15643481 | TM0718 | purine-binding chemotaxis protein | 3 |
| 15643489 | TM0726 | tldD protein | 5 |
| 15643490 | TM0727 | pmbA-related protein | 2 |
| 15643550 | TM0787 | ribulose-1,5-biphosphate synthetase | 4 |
| 15643639 | TM0877 | phosphopyruvate hydratase | 7 |
| 15643674 | TM0912 | basic membrane protein | 4 |
| 15643685 | TM0923 | hypothetical protein | 2 |
| 15643711 | TM0949 | LacI family transcription regulator | 2 |
| 15643772 | TM1014 | hypothetical protein | 18 |
| 15643885 | TM1128 | ferritin | 2 |
| 15643887 | TM1130 | phosphate butyryltransferase | 3 |
| 15643892 | TM1135 | branched chain amino acid ABC transporter periplasmic amino acid-binding protein | 2 |
| 15643897 | TM1140 | hypothetical protein | 3 |
| 15643936 | TM1180 | hypothetical protein | 2 |
| 15643960 | TM1204 | maltose ABC transporter periplasmic maltose-binding protein | 2 |
| 15644032 | TM1276 | sugar ABC transporter ATP-binding protein | 5 |
| 15644152 | TM1400 | aspartate aminotransferase | 3 |
| 15644153 | TM1401 | D-3-phosphoglycerate dehydrogenase | 2 |
| 15644170 | TM1419 | myo-inositol-1-phosphate synthase-related protein | 3 |
| 15644184 | TM1433 | oxidoreductase | 2 |
| 15644250 | TM1502 | elongation factor Tu | 2 |
| 15644358 | TM1610 | F0F1 ATP synthase subunit beta | 4 |
| 15644361 | TM1613 | F0F1 ATP synthase subunit delta | 2 |
| 15644376 | TM1628 | ribose-phosphate pyrophosphokinase | 2 |
| 15644400 | TM1652 | hypothetical protein | 3 |
| 15644468 | TM1721 | hypothetical protein | 37 |
| 15644475 | TM1729 | outer membrane protein | 15 |
| 15644529 | TM1785 | acetylornithine aminotransferase | 4 |
| 15644545 | TM1801 | hypothetical protein | 3 |
| 15644553 | TM1809 | hypothetical protein | 2 |
| 15644582 | TM1839 | maltose ABC transporter periplasmic maltose-binding protein | 31 |
| 15644621 | TM1878 | bifunctional UDP-sugar hydrolase/5'-nucleotidase periplasmic precursor | 4 |
